# Supplementary material for: Re-exposure to reward re-evaluates related memories
Source: Curr Biol. 2026 Feb 2;36(3):565–575.e3. doi: 10.1016/j.cub.2025.11.058 (PMC12881916; doi:10.1016/j.cub.2025.11.058)
Supplement: Document S1. Figures S1–S4 [file mmc1.pdf]

Current Biology, Volume *36*

Supplemental Information

Re-exposure to reward

re-evaluates related memories

Carolin Warnecke, Johanna A. Schweizer, Benedetta Zattera, Dennis Goldschmidt, Kerstin Leptien, and Johannes Felsenberg

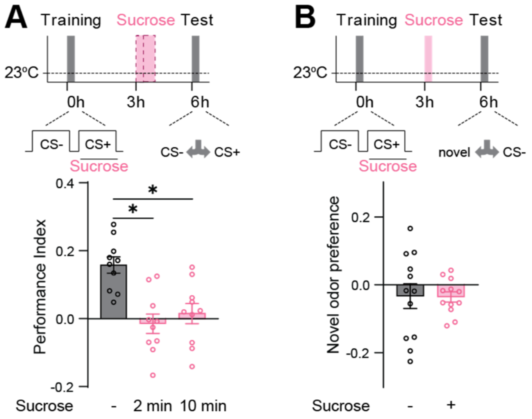

<u>Figure S1:</u> Reward re-exposure specifically diminishes expression of olfactory reward memory (related to Figure 1). A) Exposing flies to sucrose for 2 min or 10 min at 3 h after sucrose learning leads to diminished 6-h memory performance (*n* ≥ 9). B) Flies show no approach behavior to the CS- when given the choice between the CS- and a novel odor in a 6-h memory test, independently of whether they have been re-exposed to sugar 3 h after training or not (*n* ≥ 10). Asterisks denote significant differences ($p < 0.05$, ANOVA)

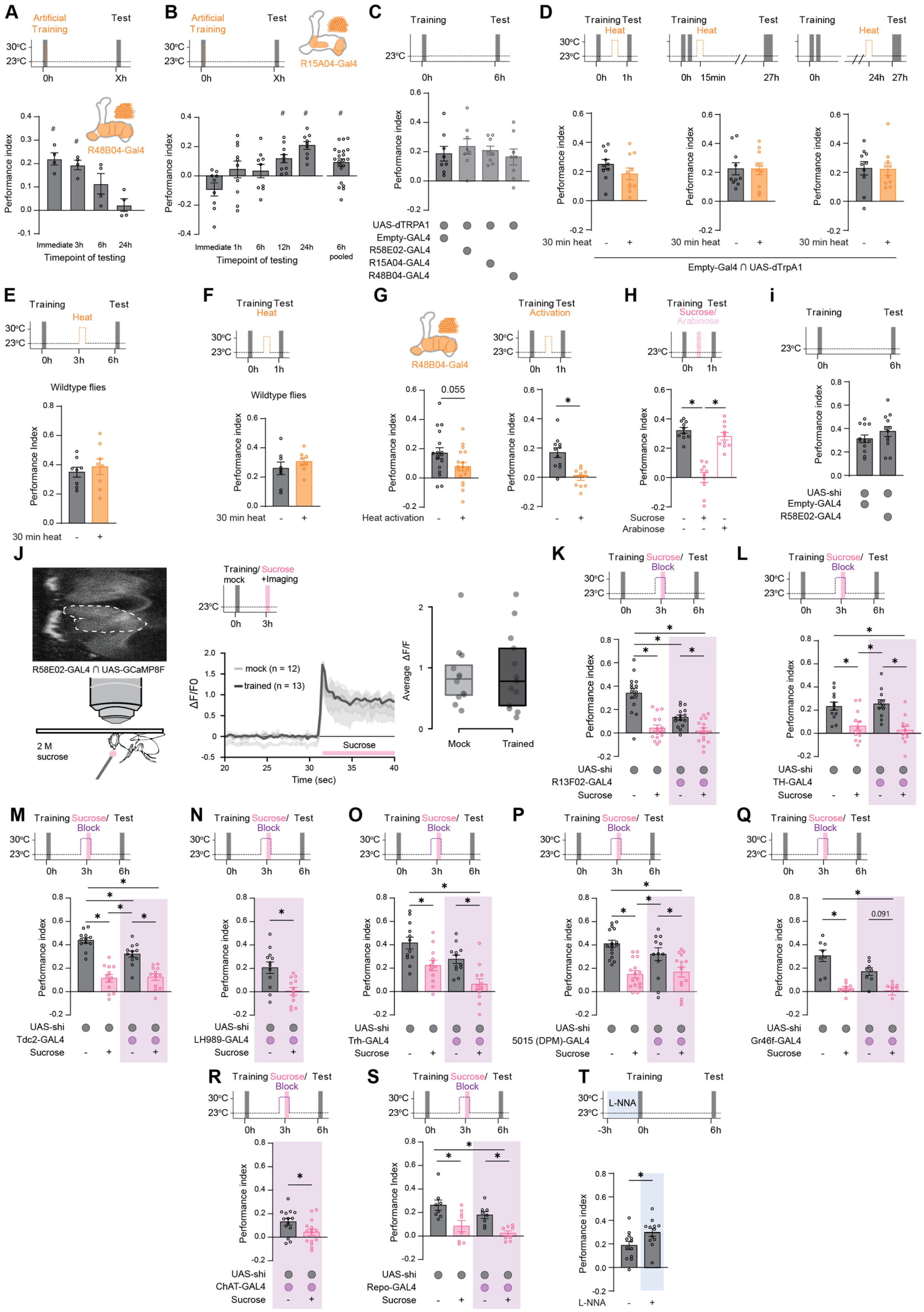
A
Artificial Training
Test
30°C
23°C
0h
Xh
R48B04-Gal4
Performance index
Immediate 3h 6h 24h
Timepoint of testing
B
Artificial Training
Test
R15A04-Gal4
Immediate 1h 6h 12h 24h 6h pooled
Timepoint of testing
C
Training
Test
UAS-dTRPA1
Empty-GAL4
R58E02-GAL4
R15A04-GAL4
R48B04-GAL4
D
Training Test
Heat
0h 1h
15min
27h
24h
30 min heat
Empty-Gal4 ∩ UAS-dTrpA1
E
Wildtype flies
Performance Index
F
G
R48B04-Gal4
Activation
0.055
Heat activation
H
Sucrose/ Arabinose
Sucrose
Arabinose
i
UAS-shi
Empty-GAL4
R58E02-GAL4
J
R58E02-GAL4 ∩ UAS-GCaMP8F
2 M sucrose
Training/ mock
Sucrose +Imaging
ΔF/F0
mock (n = 12)
trained (n = 13)
Time (sec)
Average ΔF/F
Mock
Trained
K
Training Sucrose/ Block Test
R13F02-GAL4
L
TH-GAL4
M
Tdc2-GAL4
N
LH989-GAL4
O
Trh-GAL4
P
5015 (DPM)-GAL4
Q
Gr46f-GAL4
0.091
R
ChAT-GAL4
S
Repo-GAL4
T
L-NNA
-3h 0h 6h

<u>Figure S2:</u> Activity of reward DANs bidirectionally influences sugar memory retrieval and sucrose-induced memory re-evaluation does not depend on PAM dopaminergic or other tested neural populations (related to Figure 2). A) Training with R48B04-GAL4 labeled neurons implements an appetitive STM (*n* =4). B) Artificial training with R15A04-GAL4 labeled neurons exclusively leads to appetitive LTM expression. A 6-h memory was not expressed in this data set. However, pooling 6-h memory performance with datapoints from other data sets shows robust memory expression (last bar "6 h pooled") (*n* ≥ 9). C) Flies expressing *UAS-TRPA1* in R58E02- GAL4, R15A04-GAL4 and R48B04-GAL4 labeled neurons show comparable performance in sucrose learning as controls crossed to empty-GAL4 (*n* ≥ 8). D) Flies expressing an UAS-TRPA1 together with an empty-GAL4 show no difference in memory performance when exposed to heat in the STM, LTM with early activation or LTM with late activation scheme (*n* ≥ 8). E) Exposing wild-type flies (wt, Canton-S) to 30 min heat 3 h after sucrose learning does not affect retrieval at the 6-h memory test (*n* ≥ 8). F) Exposing wt flies to heat for 30 min at 15 min after sucrose training does not influence 1-h memory retrieval (*n* ≥ 8). G) Activation of STM DANs 15 min after learning leads to diminished 1-h memory retrieval. The two graphs show the separate data of two repetitions of the same experiment. Combined data shown in Fig. 2D. H) Re-exposure to sucrose for 5 min at 30 min after sucrose training leads to diminished 1-h memory performance. Following the same timeline post-training exposure to arabinose has no effect on memory performance (*n* ≥ 8). I) Flies expressing shi^{ts} driven by R58E02-GAL4show similar memory performance after sucrose training compared to genetic controls (*n* ≥ 10). J) GCaMP8f expression in projection of PAM DANs under control of R58E02 driver. ROI depicted by dotted line. 3 h post training 2 M sucrose was offered to the proboscis of trained and mock trained flies placed under 2-photon microscope. Calcium signal was measured in the tip of the horizontal MB lobe. The averaged calcium signal in PAM dendrites in response to sucrose stimulation does not differ between trained and mock trained flies. K-S) Blocking any of the tested neuronal population during sucrose re-exposure does not impede sucrose-mediated memory devaluation. Candidates tested: KCs (R13F02-GAL4) (*n* ≥ 15) (K), PPL1-, PPL2-, PAL-, PPM1-3-DANs (TH-GAL4) (*n* ≥ 10) (L), octopaminergic neurons (Tdc2-GAL4) (*n* ≥ 12) (M), PD2a1/b1 LHONs (LH989-GAL4) (*n* ≥ 11) (N), serotonergic neurons (Trh-GAL4) (*n* ≥ 11) (O), pair of DPM-neurons (5015-GAL4) (*n* ≥ 12) (P), sweet gustatory receptor neurons (Gr64f-GAL4) (*n* ≥ 7) (Q), cholinergic neurons (ChAT-GAL4) (*n* ≥ 14) (R) and glia cells (repo-GAL4) (*n* ≥ 10) (S). T) Feeding L-NNA for 3 h before training to inhibit NOS activity leads to increased memory retrieval at a 6-h test (*n* ≥ 8). In all figures, data represent the mean ± s.e.m. Individual n are indicated by circles. Asterisks denote significant differences ($p < 0.05$, *t*-test or ANOVA). Asterisks denote significant differences to 0 ($p < 0.05$, one sample *t*-test) in A&B and unpaired *t*-test ($p < 0.05$) in G.

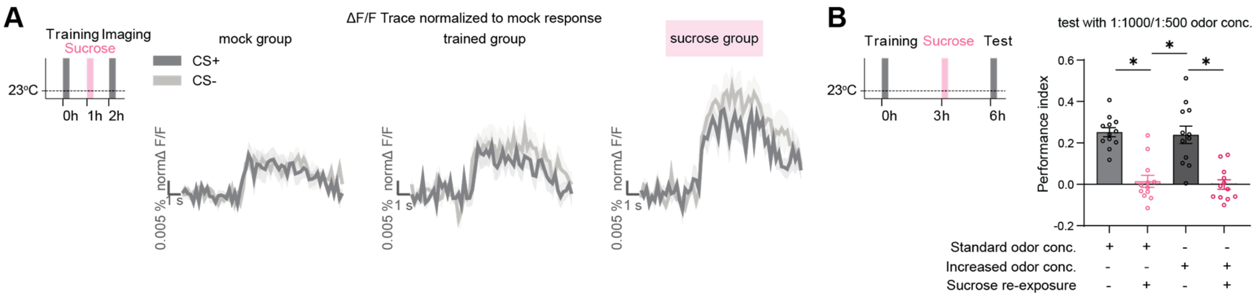

<u>Figure S3:</u> Memory trace is intact after US-re-exposure (related to Figure 3). A) Flies expressing GCamp7s in VT1211-Gal4 labeled neurons are sugar trained in T-mazes and re-exposed to sucrose in housing vials 1 h later, 2 h after learning calcium signals were measured in MBON-β'2mp and MBON-γ5β'2a neurons. Here, ΔF/F0 traces normalized to the average odor response of the corresponding odor of the mock are shown for the respective groups (see Methods). B) 6-h memory was tested after 5 min re-exposure to sucrose reward across different odor concentrations in the test (*n* ≥ 11). Flies were trained with an odor concentration of $\sim 10^{-3}$ and tested with either the same concentration or a higher odor concentration ($2 \times \sim 10^{-3}$). Asterisks denote significant differences ($p < 0.05$, ANOVA).

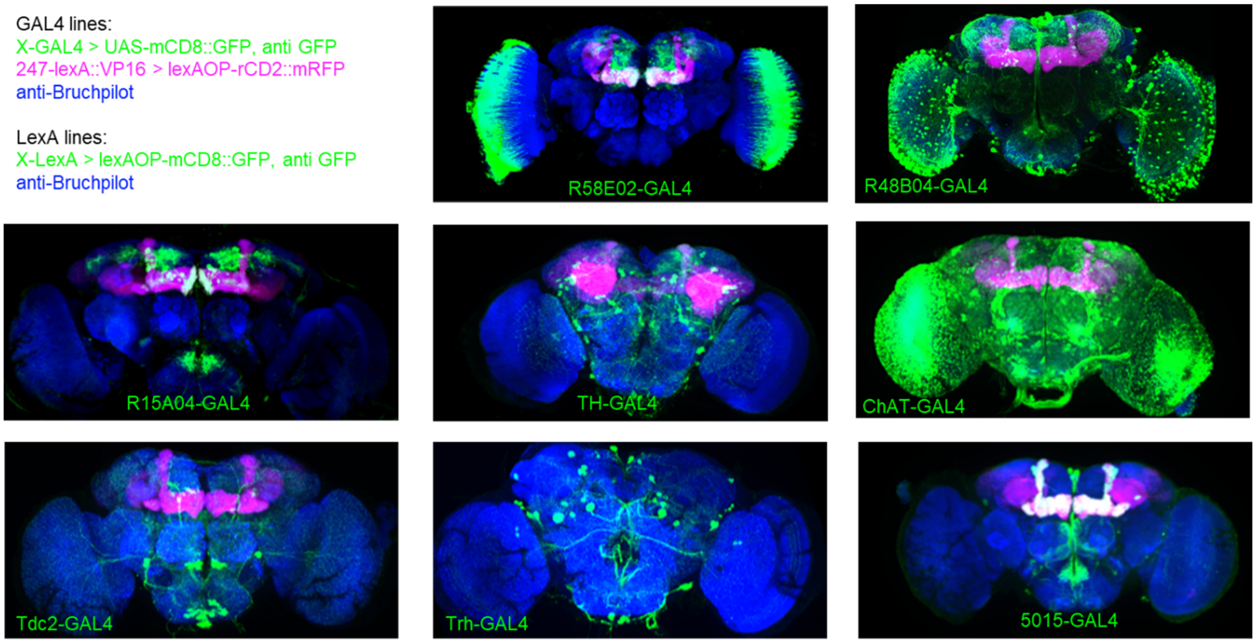

<u>Figure S4:</u> GAL4 expression patterns (related to Figure 2 and S2).
