# Supplementary material for: Re-exposure to reward re-evaluates related memories
Source: Curr Biol. 2026 Feb 2;36(3):565–575.e3. doi: 10.1016/j.cub.2025.11.058 (PMC12881916; doi:10.1016/j.cub.2025.11.058)
Supplement: Document S2. Article plus supplemental information [file mmc2.pdf]

Current Biology

Article

Re-exposure to reward re-evaluates related memories

Highlights

- Re-exposure to reward devalues multiple related olfactory memories
- Active forgetting and reward-induced memory devaluation are separable processes
- Devaluation of the reward memory leaves memory traces intact
- Contextual information and new learning constrain memory devaluation

Authors

Carolin Warnecke,
Johanna A. Schweizer,
Benedetta Zattera,
Dennis Goldschmidt, Kerstin Leptien,
Johannes Felsenberg

Correspondence

johannes.felsenberg@fmi.ch

In brief

Warnecke et al. show that re-exposure to reward alone diminishes multiple associated olfactory memories. This devaluation is reward specific and independent of active forgetting pathways. The authors find that contextual information and new learning constrain memory devaluation, suggesting an intricate relationship between learned information and the revaluation of specific memories.

Warnecke et al., 2026, Current Biology *36*, 565–575
February 2, 2026 © 2025 The Authors. Published by Elsevier Inc.
https://doi.org/10.1016/j.cub.2025.11.058

CellPress

Current Biology

CellPress
OPEN ACCESS

Article

Re-exposure to reward re-evaluates related memories

Carolin Warnecke,[1,2] Johanna A. Schweizer,[1,2] Benedetta Zattera,[1,2] Dennis Goldschmidt,[1] Kerstin Leptien,[1] and Johannes Felsenberg[1,3,*]
[1]Friedrich Miescher Institute for Biomedical Research, Fabrikstrasse 24, 4056 Basel, Switzerland
[2]University of Basel, Petersplatz 1, 4001 Basel, Switzerland
[3]Lead contact
*Correspondence: johannes.felsenberg@fmi.ch
https://doi.org/10.1016/j.cub.2025.11.058

SUMMARY

To adapt behavior in changing environments, animals must continuously re-evaluate previously learned associations. This flexibility of memory systems has been identified as a promising strategy to target maladaptive memories. Here, we show that re-exposure to an unconditioned stimulus (US) alone, a sugar reward, can re-evaluate appetitive memories in *Drosophila melanogaster*. Using olfactory conditioning, we demonstrate that unpaired US exposure after memory formation reduces conditioned responses to multiple odor-reward associations. This reduction is specific to the re-exposure of the trained US and does not result from an altered motivational state or generalized behavioral suppression. Importantly, this US-induced memory devaluation engages mechanisms distinct from dopamine-driven modulation of memory accessibility, indicating a separate process of memory re-evaluation. Moreover, we find that sugar re-exposure diminishes both short- and long-term memory phases and can act on consolidated memories, suggesting broad temporal applicability. Notably, this devaluation does not change the reward-memory trace in specific mushroom body output neurons, implying that the underlying memory trace remains intact despite behavioral suppression. Our findings reveal a mechanism by which reward re-experience pervasively devalues associated memories, offering a potential approach to target multiple memories without requiring re-exposure to individual cues. This work provides insight into how experience can broadly reshape memory networks and may inform future approaches for persistent memory modification.

INTRODUCTION

To adapt to changing environments, animals continuously have to re-evaluate learned behavior. Understanding the underlying mechanism has the potential to support approaches to modify maladaptive memories in humans.[1–3] Re-exposure to cues in the absence of an expected outcome can lead to a change in previously learned behavior. However, these changes are limited to a specific cue-outcome (e.g., reward) association and, in most cases, are not permanent.[4] Developing protocols that induce persistent changes in memory accessibility across multiple retrieval cues is crucial for targeted treatment of maladaptive memories. Here, we explore how the re-exposure to a reward alone, in the absence of predictive cues, can affect associated memories.

Classical conditioning provides a robust experimental framework for investigating how memories are formed and subsequently modified. In this paradigm, one or more sensory cues (conditioned stimuli [CS]) are paired with a biologically significant event (unconditioned stimulus [US]), such as rewarding or harmful stimuli, leading to the formation of an associative memory.[5] When tested shortly after training, a conditioned response to the CS is expressed, reflecting short-term memory (STM). For these memories to persist and influence behavior over extended periods (long-term memories [LTMs]), they have to be consolidated during the first hours after learning.[6] Disrupting this consolidation phase, e.g., by pharmacological or behavioral perturbation, prevents the formation of LTM.[6,7] Once consolidated, memories are resistant to interference. However, when a consolidated memory is retrieved but the outcome deviates from the original learning experience (prediction error), e.g., the CS is not followed by the US, learned behavior can be modified. The magnitude of the prediction error plays a critical role in determining the extent of memory change. For instance, a brief presentation of the CS without the US (small prediction error) can trigger memory destabilization and a second round of consolidation (reconsolidation).[8–10] In contrast, multiple or prolonged presentations of the CS without the US (large prediction error) promote extinction learning, resulting in the formation of an opposing "CS no-US" memory.[4,5,8] Although these approaches focus on altering memories through re-exposure to a specific cue, the CS, they may not capture the full complexity of memory retrieval when multiple sensory cues are involved. To target memories across multiple associations, it has been suggested that re-exposure to the US alone can facilitate the re-evaluation of multiple associated cues.[11–13] However, the precise conditions and neuronal processes that allow such a US-driven re-evaluation remain poorly understood.

© 2025 The Authors. Published by Elsevier Inc.
This is an open access article under the CC BY license (http://creativecommons.org/licenses/by/4.0/).

We investigated how re-exposure to the US, in this case the sucrose reward, modulates learned responses in *Drosophila melanogaster*. In our paradigm, flies learn to associate an odor (CS+) that is paired with sucrose (US) during the training, resulting in a preference for the CS+ over an odor that was presented alone during training (CS−).[14,15] Crucial parts of these olfactory memories are stored in the mushroom body (MB) as dopamine-driven changes between odor-coding Kenyon cells (KCs) and specific MB output neurons (MBONs).[16,17] Within the MB, STM and LTM are established in parallel pathways that require reinforcement from distinct populations of dopaminergic neurons (DANs): STM-DANs and LTM-DANs.[18–28] Memory traces (or engrams) that support learned approach behavior manifest as odor-specific changes in the activity of corresponding MBONs.[29,30] Beyond their role in reinforcement during learning, DANs have diverse functions in memory-related processes, including state-dependent modulation of the MB output system, which can regulate memory accessibility.[31–38] Using appetitive olfactory conditioning while leveraging genetic access to specific cells of the fly memory center, we demonstrate that re-exposure to the sugar reward effectively devalues reward-associated olfactory memories in a manner that is both context dependent and applicable across different memory phases. Although activating distinct subsets of DANs can produce a similar reduction in learned behavior, our findings indicate that US re-exposure-induced devaluation and dopamine-driven memory modulation are mechanistically distinct processes. Together, we show that US re-exposure leads to memory re-evaluation across multiple memories.

RESULTS

Reward-specific re-exposure diminishes multiple associated memories

Re-exposure to a memory-related stimulus can lead to an update of learned associations. In memory extinction, learned responses temporarily decline when the CS+ is repeatedly presented without the US.[4] However, the consequences of re-experiencing the US alone after conditioning are less well understood. To test the effects of US re-exposure on reward memory, we utilized differential olfactory conditioning using sucrose as the appetitive US in a classical T-maze setting.[14,15] Three hours after a single olfactory conditioning trial, flies were re-exposed to the US alone in housing vials for 5 min or transferred to similar vials without the US as a control. Compared with controls, re-exposing flies to the US reduced learned approach behavior in a binary choice between the CS+ and the CS− at 6 and 27 h after training (Figures 1A and 1B). The duration of the re-exposure appeared to be less crucial, as shorter (2 min) or longer (10 min) US re-exposures produced similar reductions in memory expression (Figure S1A). Further, exposing flies to the US 24 h after training, when memory is consolidated, reduced memory expression (Figure 1B). In contrast, the same US exposure 3 h before training left memory retrieval unchanged (Figure 1C), demonstrating that it is not mere US exposure but the post-training experience that reduces learned behavior. In binary choice tests, the lack of learned behavior can arise from either a decrease in the CS+ approach or an increased attraction to the CS−. To explore these possibilities, we modified the memory test to compare choices between the CS+ and a novel odor and separately between the CS− and a novel odor. These tests revealed that, rather than an altered response to the CS−, the reduction in memory expression is specifically due to a diminished approach to the CS+ (Figures 1D and S1B). Thus, re-exposure to the US after conditioning reduces odor-specific learned approach behavior. Next, we tested whether this reduction is specific to the reward used as US during training. Intriguingly, exposure to standard fly food instead of the US did not alter memory expression (Figure 1E), indicating that changes in the learned response are independent of changes in general hunger levels. Further, the inability of standard food to reduce the learned approach suggests that memory loss is a specific consequence of the re-exposure to the trained US. To further test this specificity, we used a sugar that differs in caloric value. Previous work has shown that flies can distinguish between sugars with or without caloric content within minutes.[39] Thus, we tested whether the re-exposure to the non-caloric but sweet-tasting sugar arabinose alters memory expression. We found that re-exposure to arabinose left sucrose-memory expression unaltered (Figure 1F), supporting the idea that re-evaluation is US specific. Together, these experiments suggest that re-exposure to the appetitive US used during training specifically diminishes responses to the associated CS+. The specificity in reducing learned behavior suggests that the re-exposure should affect all associations formed with a particular US. To assess whether US-induced changes in reward memory extend to multiple learned associations, we trained flies in two consecutive trials using two different odor pairs (CS−1/CS+1 and CS−2/CS+2). Flies successfully learned and retrieved both memories (Figures 1G and 1H). However, when sucrose was presented 3 h after the second training session, retrieval of both memories was abolished (Figures 1G and 1H). These results demonstrate that memory reduction via sucrose re-exposure affects multiple CS+ odors associated with the sucrose reward.

Together, these findings show that the re-exposure to the US alone devalues multiple related reward memories.

PAM-DAN activity and reward re-exposure independently modulate memory devaluation

Post-training re-exposure to the US, the sucrose reward used during conditioning, diminished reward-memory expression. Sugar reward is encoded through the activity of DANs from the PAM cluster, and artificial activation of these neurons can substitute for the natural reward during training.[23,24] Activation of distinct subgroups of PAM-DANs can either induce STM that decays quickly or generate LTM that lacks a STM component (Figures S2A and S2B).[20–22,38,40] Beyond their role in memory acquisition, DANs have also been implicated in both the updating and forgetting of associative memories.[31,38,41–44] Therefore, we investigated how PAM-DANs contribute to US-mediated memory devaluation. In line with previous studies,[31,38,44] we found that thermogenetic activation of PAM-DANs (R58E02-GAL4) for 30 min at 3 h after training leads to loss-of-memory performance at a 6-h test (Figure 2A). Further, activation of PAM-DANs 15 min after training diminished both STM (1-h test) and LTM (27-h test), and activation at 24 h after training also reduced 27-h memory performance (Figures 2B and 2C). Importantly, repeating experiments at room temperature or

CellPress
OPEN ACCESS

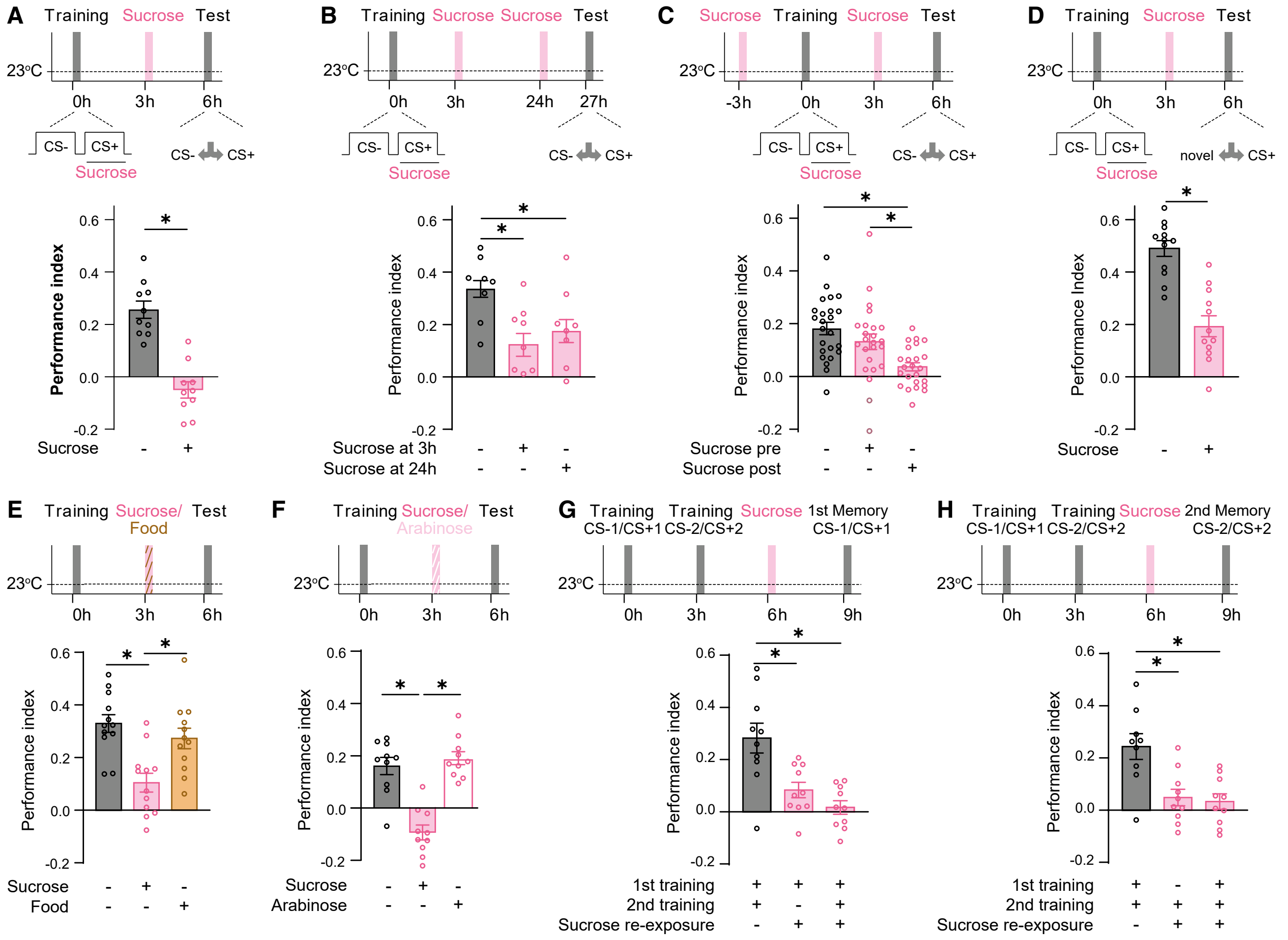

Figure 1. Reward re-exposure diminishes the expression of olfactory reward memory

(A) Re-exposing flies to the sucrose reward (US) for 5 min at 3 h after training leads to a reduction of 6-h memory performance ($n \geq 10$).

(B) Re-exposing flies to the sucrose at 3 or 24 h after training leads to diminished memory retrieval in a 27-h test ($n \geq 10$).

(C) Exposing flies to sucrose 3 h before training does not affect 6-h memory ($n \geq 25$).

(D) In a test against a novel odor, the approach to the CS+ is reduced after sucrose re-exposure ($n \geq 12$).

(E) Although re-exposing flies to sucrose for 5 min at 3 h after training leads to memory devaluation, feeding flies regular fly food instead does not affect 6-h memory performance ($n \geq 11$).

(F) Re-exposing flies to sweet but non-caloric sugar arabinose for 5 min 3 h after training does not reduce memory performance at 6 h ($n \geq 12$).

(G and H) Sucrose re-exposure after two sets of training using different odor sets leads to the devaluation of both memories ($n \geq 10$).

In all figures, data represent the mean ± SEM. Individual *n* indicated by circles. Asterisks denote significant differences ($p < 0.05$, *t* test or ANOVA).

See also Figure S1.

exposing genetic controls as well as wild-type flies to elevated temperatures (used for thermogenetic activation) did not affect reward-memory retrieval (Figures S2C–S2F). Thus, broad activation of PAM-DANs leads to a lasting loss of reward memory, independent of whether activation occurs during or after the memory consolidation phase window.

Given the specificity of PAM-DANs subgroups for STM and LTM formation, we next asked whether these subgroups could selectively perturb their respective memory phase. Consistent with their established role in STM formation, we found in two independent experiments that thermogenetic activation of STM-associated DANs (R48B04-GAL4) reduced STM but left LTM intact (Figures 2D and S2G). In contrast, the activation of LTM-associated DANs (R15A04-GAL4) affected both STM and LTM stability (Figures 2E and S2D). Interestingly, activating either STM- or LTM-DANs at 3 h post training did not alter memory retrieval at 6 h (Figures 2F and S2C). This lack of effect may reflect the presence of two parallel memory components at this time point, such that loss of one is compensated by the other. Alternatively, a third PAM-DAN population might be required to modulate the memory phase between 3 and 6 h. For early memory phases, it is known that activation of STM-DANs or training with certain sweet-only sugars leads exclusively to a short-lasting STM.[39,45] Accordingly, we tested whether arabinose exposure after sucrose training can specifically devalue STM. However, exposing sucrose-trained flies to arabinose 30 min after training left 1-h STM intact, confirming that memory devaluation is US specific (Figure S2H). This result

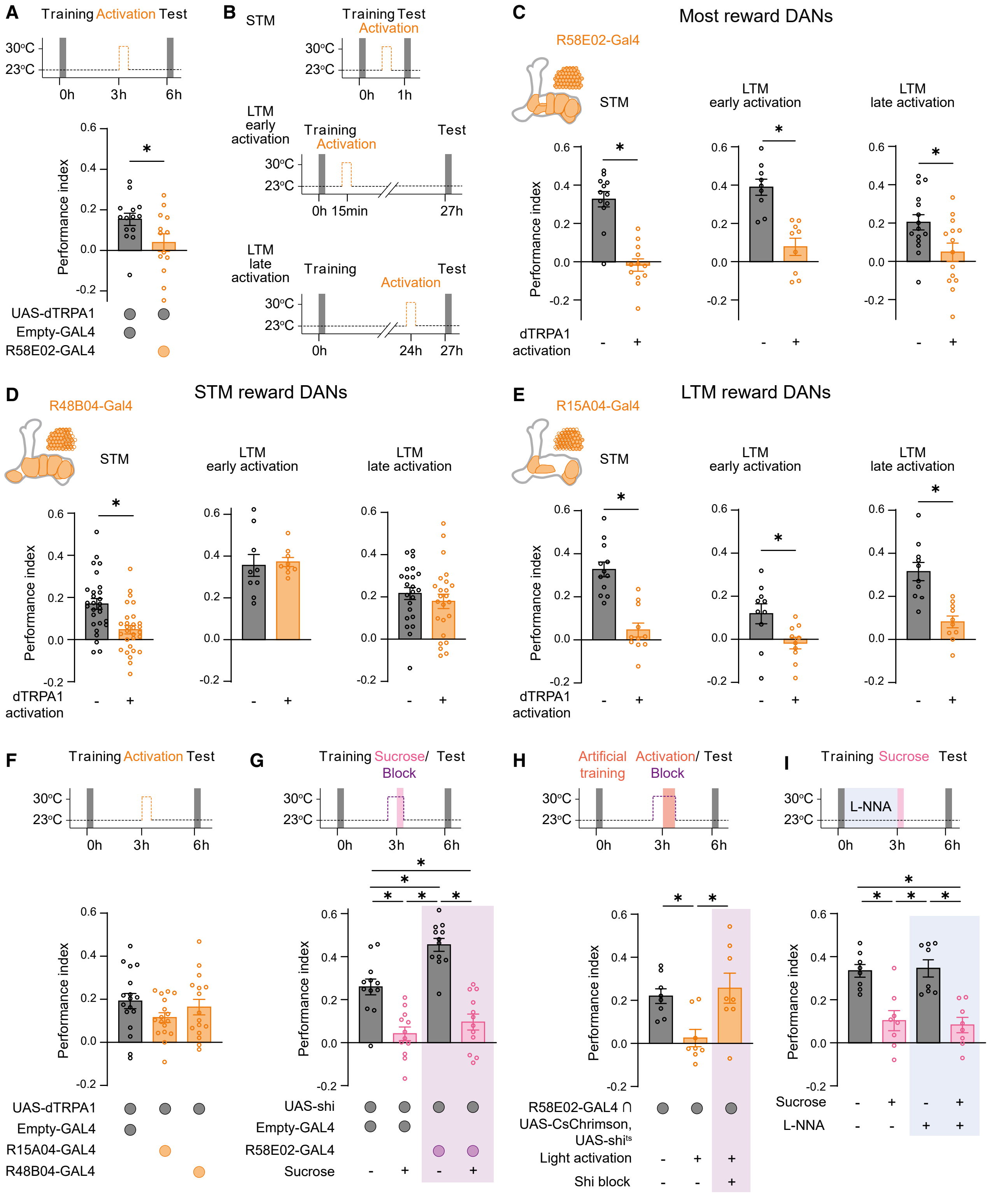

Figure 2. Activity of reward DANs bidirectionally influences sugar-memory retrieval

(A) Activating PAM-DANs 3 h after sucrose training reduces 6-h memory retrieval ($n \geq 12$).

(B) Depiction of the different time protocols.

(C) Artificially activating PAM-DANs (R58E02-GAL4) 15 min after training reduces 1-h memory performance. Likewise, activating PAM-DANs for 30 min at 15 min or 24 h after sucrose training diminishes 27-h memory ($n \geq 9$).

(legend continued on next page)

further suggests that arabinose exposure does not mimic activation of STM-DANs, hinting at separable mechanisms underlying memory devaluation. Altogether, while these data reveal a complex interaction between memory phases and vulnerability to dopamine-driven memory loss that might or might not be linked, the mechanistic link between US re-exposure and PAM-DAN activity remains unresolved.

To explore the link between PAM-DAN activation and US-mediated devaluation of reward memories, we investigated whether blocking the function of PAM-DANs affects the US re-exposure-mediated memory devaluation. Using the temperature-sensitive dynamin mutant *shibire*^{ts} (*shi*^{ts}),[46] we blocked vesicle release from PAM-DANs during post-training US re-exposure. Surprisingly, blocking PAM-DANs during re-exposure did not affect US-mediated memory devaluation (Figure 2G), suggesting that the effect of US re-exposure is independent of PAM-DAN output. However, blocking PAM-DANs without US re-exposure led to increased memory performance (Figures 2G and S2I). This facilitation might suggest that ongoing vesicle release from PAM-DANs restricts reward-memory persistence or accessibility, potentially similar to dopamine-driven active forgetting of aversive memories.[31] Because blocking vesicle release from PAM-DAN does not affect US-mediated memory devaluation but PAM-DAN activation diminished reward memory, we aimed to test whether vesicle release is required for PAM-DAN-activation-mediated memory loss. Flies that co-express the light-gated cation channel *CsChrimson* and *shi*^{ts} in R58E02-GAL4-labeled neurons, to activate PAM-DANs while blocking their vesicle release, fail to learn from sugar (data not shown). However, substituting sugar reward with activation of the labeled PAM-DANs leads to appetitive 6-h memory. Like sucrose-reward memory, reactivation of PAM-DANs for 30 min at 3 h after the artificial training diminished 6-h memory. However, blocking vesicle release during the reactivation of PAM-DANs prevented such memory loss (Figure 2H). Thus, although we were only able to investigate artificial memory, these data suggest that PAM-DAN-induced memory loss requires vesicle output. In contrast, sucrose-US-induced devaluation does not. To further probe this separation, we expressed GCaMP8f in R58E02-GAL4-positive neurons to assess sucrose responses in PAM-DANs of trained and mock-trained flies. In line with the functional separation of the two processes, we found no difference in the sucrose responses between trained flies and those receiving mock treatment (Figure S2J). These findings indicate that US-driven devaluation and DAN-driven memory loss occur through distinct mechanisms.

To investigate which circuit components are involved in reward-memory devaluation, we expressed *shi*^{ts} in other neurons of the MB-network, including KCs (R13F02-GAL4), non-PAM DANs (TH-GAl4), octopaminergic neurons (Tdc2-GAL4), PD2a1/b1 lateral horn output neurons (LH989-GAL4), serotonergic neurons (Trh-GAL4), DPM neurons (5015-GAL4), sweet-sensing gustatory receptor neurons (Gr64f-GAL4), most cholinergic neurons (ChAT-GAL4), and glia cells (repo-GAL4) (Figures S2K–S2S). However, blocking the output of these neurons did not prevent US-induced memory devaluation. Finally, we tested the involvement of nitric oxide (NO), a known co-transmitter of DANs. Consistent with previous findings in aversive memory,[40] blocking NO synthase (NOS) during training using L-NNA increased sucrose-memory performance (Figure S2T). This effect was specific to the learning phase and not due to altered consolidation, as feeding L-NNA after learning did not affect memory (Figure 2I). However, blocking NOS during US re-exposure did not prevent memory devaluation (Figure 2I).

Together, our findings imply that US-induced memory devaluation operates independently of PAM-DAN dopamine and NO signaling, suggesting the involvement of other, yet-to-be-identified elements of the memory circuit. However, PAM-DAN vesicle release, as well as NO signaling in the MB, appear to restrict the persistence or accessibility of reward memories, indicating that multiple parallel systems regulate access to reward memories.

Re-exposure-driven memory devaluation leaves sugar-memory trace intact

The reduction of memory expression following unpaired US re-exposure could result either from the loss of the memory trace itself or from interference via parallel circuits. Previous work has indicated that reward memories at 2 h after training are established as reduced odor-driven activation of avoidance-coding MBONs in the β′2 compartment.[29] To explore whether this memory trace is maintained after sucrose exposure, we expressed the genetically encoded calcium sensor GCaMP7s[47] in MBON-β′2mp and MBON-γ5β′2a (VT1211-GAL4). In the T-maze, flies were subjected either to training or to a mock-training protocol (odor exposure without sucrose reward). Trained flies were then divided into two groups: one group was re-exposed to the US alone 1 h after training, while the other underwent handling without sucrose. From each of the 3 groups, some flies were used for odor-response measurements 2 h after training (Figure 3A), and the remaining flies were tested behaviorally for memory performance. In the behavioral test, trained flies showed learned approach behavior, whereas US re-exposure

(D) Activating STM-DANs (R48B04-GAL4) 15 min after training leads to memory reduction in a 1-h memory test (data from two independent experiments; Figure S2D), whereas activating STM-DANs 15 min or 24 h after sucrose training leaves 27-h memory unaltered ($n \geq 9$).

(E) Activation of LTM-DANs labeled by R15A04-GAL4 15 min after training diminishes 1-h memory retrieval. Likewise, activating LTM-DANs 15 min or 24 h after sucrose training lowers 27-h memory ($n \geq 10$).

(F) When LTM- or STM-DANs are activated 3 h after sucrose training, 6-h memory retrieval stays intact ($n \geq 16$).

(G) When blocking R58E02-GAL4-labeled neurons (using *shi*^{ts}) during sucrose re-exposure, sucrose-mediated memory devaluation still occurs ($n \geq 12$). Only blocking PAM-DANs for 30 min at 3 h after training enhances 6-h memory expression ($n \geq 12$).

(H) When output from PAM-DANs is restricted while artificially reactivating neurons at 3 h for 30 min, DAN activity-mediated memory reduction is impaired ($n \geq 6$).

(I) Blocking NOS activity between training and sucrose re-exposure does not impact sucrose-mediated memory devaluation ($n \geq 8$).

In all figures, data represent the mean ± SEM. Individual *n* indicated by circles. Asterisks denote significant differences ($p < 0.05$, *t* test or ANOVA).

See also Figure S2.

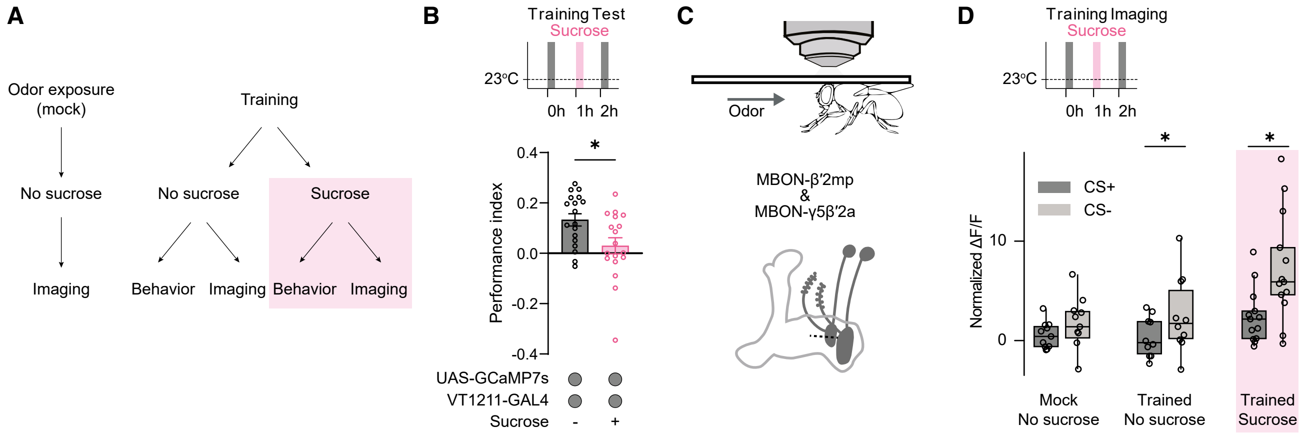

Figure 3. Memory trace is intact after US-re-exposure

(A) Scheme of experimental procedure.

(B) Re-exposure to sucrose 1 h after learning leads to diminished memory in a 2-h test ($n \geq 18$).

(C) Illustration of the setup for head-fixed flies under two-photon microscope (top). Depiction of MBON-β′2mp and MBON-γ5β′2a neurons (bottom).

(D) Calcium responses in MBON-β′2mp and MBON-γ5β′2a at 2 h after training show differences between the CS− and the CS+ in trained flies but not in flies receiving mock treatment. These differences in the responses are still present when flies are re-exposed to the sucrose reward 1 h after training. Normalized peak responses to the paired and unpaired odors are shown. Peak responses are normalized to the group average of the mock group for that odor and to the response to the air puff (STAR Methods) ($n \geq 9$)

Shown is the mean ± SEM. Individual *n* indicated by circles. Asterisks denote significant differences ($p < 0.05$, *t* test).

See also Figure S3.

diminished this memory expression (Figure 3B). Consistent with previous findings, we find that trained, but not mock-treated, flies showed a subtle but significant reduction in normalized responses to the CS+ compared with the CS− (Figures 3C and 3D). Re-exposure to the US 1 h after training appears to increase the responses to both odors. However, despite this elevated overall activity and the reduction in behavioral memory expression across different odor concentrations, flies in the re-exposed group still exhibited a differential response between the CS+ and CS− (Figures 3D, S3A, and S3B). Therefore, we conclude that US-mediated devaluation does not erase the initial memory trace but instead operates through other mechanisms.

Contextual control of memory devaluation

Contextual cues are known to influence memory retrieval.[48] However, whether memory update processes depend on contextual information is poorly understood. In our standard protocol, re-exposing flies to sucrose-reward (US) in housing vials reduced memory scores. In contrast, in the retraining experiments, US re-exposure during the second training in the T-maze did not affect the previously acquired memory (Figures 1G and 1H). To test whether these differences arise from new learning or differences in the context, we re-exposed flies 3 h after training to the US alone in the T-maze training chambers or in housing vials. Interestingly, we found that US re-exposure in the T-maze chambers left the reward memory unchanged, whereas re-exposure in the standard protocol again diminished the learned approach (Figure 4A). These findings suggested that re-exposure to the US in the training context prevents memory loss. To test this idea, we performed experiments where flies were trained in housing vials rather than in the T-maze (for details, see STAR Methods). However, when flies trained in housing vials were re-exposed to the US in the same vials, memory expression was still reduced in the test (Figure 4B). These findings show that the contextual information that gates re-exposure-related devaluation is not linked to the experience during training. Interestingly, when we probed whether the context-dependency extends to the artificial activation of PAM-DANs after sucrose training, we found that the loss of memory due to DAN activation is context independent (Figure 4C).

Previous work has shown that novelty and new learning can prevent memory re-evaluation.[10,49] Therefore, we investigated next whether a first memory is maintained when flies receive US re-exposure in housing vials but during pairing with a novel odor that leads to new learning. Interestingly, the pairing of a novel odor (CS+2) with the US left the initial memory intact, suggesting that the formation of new associations can generally prevent memory devaluation (Figure 4D). Together, these experiments show that US-driven memory revaluation is modulated by contextual information during the re-exposure. However, what defines the boundary conditions of whether memories can be changed remains a challenging question to address in future experiments.

DISCUSSION

Our findings in *Drosophila melanogaster* reveal that re-exposure to the US, in our case the sucrose reward, robustly devalues previously acquired reward-associated olfactory memories independently of the consolidation phase. Notably, this devaluation is context dependent, suggesting that during memory re-evaluation flies integrate learned information beyond the CS+-US pairing to gate updating of learned behavior. In addition, US-exposure-driven devaluation extends across all memories linked

CellPress
OPEN ACCESS

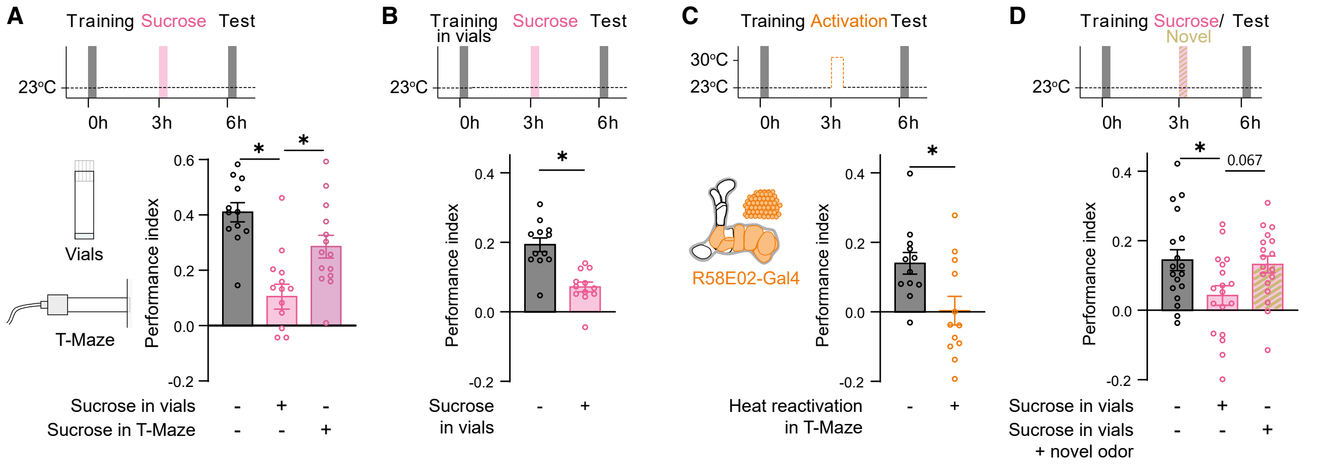

Figure 4. Sucrose-mediated memory devaluation is context dependent

(A) Sucrose memory is diminished when sucrose re-exposure is performed in housing vials. Conversely, when sucrose is presented in the T-maze context, the memory stays intact.

(B) When flies are trained in housing vials, re-exposure to sucrose in the same vials leads to memory devaluation ($n \geq 10$).

(C) Artificial reactivation of PAM-DANs in the T-maze does lead to diminished memory independent of the context ($n \geq 12$).

(D) Exposing flies to a novel odor during sucrose re-exposure in housing vials leaves the memory intact ($n \geq 18$).

In all figures, data represent the mean ± SEM. Individual *n* indicated by circles. Asterisks denote significant differences ($p < 0.05$, *t* test or ANOVA).

to that specific US, highlighting its potential as a broad mechanism for memory updating.

Reward-driven re-evaluation of appetitive olfactory memories

Previous studies have established that re-exposure to the US can modulate memories by triggering reconsolidation processes.[11,12,50] In line with these findings, our results show that re-exposure to the US allows the updating of previously acquired olfactory reward memories. As in studies with aversive US in rats,[51] the devaluation is specific to the reward used as US during training, as exposure to alternative foods sources or non-caloric sugars does not reduce sucrose memory. Moreover, our data indicate that US re-exposure can target multiple associated memories simultaneously, affecting responses to various cues associated with the US. These observations align with similar approaches using appetitive and aversive US in rodents and humans to update drug and fear-related memories across multiple CS, suggesting that the underlying principles might be similar.[13,52] However, in contrast to our findings in which the US alone is sufficient to alter memory expression, previous reports have suggested that US re-exposure induces reconsolidation without directly altering the memory.[11,12] These studies show that either pharmacological or behavioral interference is necessary to alter the targeted memories. However, our study suggests that, in principle, US re-exposure can serve as a promising alternative to memory extinction to change learned behavior permanently. Thus, future work aiming at understanding the underlying processes that enable changes of memories by US re-exposure will help to increase the efficiency of protocols that allow the updating of all memories linked to a particular event. Despite extensive experimentation, our work could not pinpoint the precise circuit components contributing to US-mediated memory devaluation. This indicates that US-induced devaluation is achieved by signaling through pathways outside the MB, via pathways not targeted by the approaches applied or through degenerate circuit motifs.

In addition to the US-driven devaluation, we explored the role of DANs, particularly those of the PAM cluster, in modulating memory expression. Although it was previously proposed that US-induced devaluation might be mediated by DAN activity,[31,38,44,53] our results indicate that the canonical output from PAM-DANs is not required for US-mediated memory devaluation. However, in line with previous studies,[44] artificial activation of PAM-DANs produces a similar loss-of-memory phenotype, although the underlying mechanisms appear to be different. Our experiments using artificial activation of subsets of PAM-DANs reveal memory-phase-specific devaluation, suggesting that distinct memory phases may be selectively vulnerable to interference through specific pathways. This is consistent with the notion that STM and LTM in the fly are stored in parallel circuits and that they can be formed and updated independently.[20,21,25–28] Though further experiments will be necessary, our data suggest that ongoing or evoked activity in distinct PAM-DANs can modulate the availability of these parallel memories specifically. For aversive experiences in flies, distinct DANs from the PPL1 cluster have been shown to drive active forgetting of weak, short-lasting memories or transient forgetting of robust LTM.[31,32] Behavioral or motivational states, e.g., hunger or thirst, can modulate ongoing or stimulus-evoked PAM-DAN activity to control odor-driven behavior and memory accessibility.[33–36,54–57] In particular, starvation-induced hunger states seem to change activity in DANs[33,35,57]; for example, satiety reduces activity in γ3-PAM-DANs, which is registered as a desirable state by flies.[54] Likewise, other post-training experience might be translated into specific patterns of ongoing PAM-DAN activity that allows to control memory accessibility based on the integration of the internal states with external cues to prioritize available learned information.

Overall, our findings provide strong evidence that US re-exposure can specifically devalue multiple reward-associated memories independently of dopamine-driven forgetting, opening new avenues for dissecting the underlying circuit and molecular mechanisms of updating learned information.

Erasure or suppression

Olfactory reward memories are encoded in the MB as changes in avoidance-coding β′2 MBONs.[29] Consistent with these studies, we observed that, 2 h after training, the responses of these MBONs to the CS+ are significantly reduced compared with the CS−, a difference not seen in mock-trained flies. It is worth mentioning that the observed changes are subtle, which aligns with previous findings using the same approach.[29] This aspect might be due to the procedure of fixing the flies for imaging experiments, as training directly under the microscope reveals more pronounced differences between CS+ and CS− in the same neurons.[55,58] However, though re-exposing flies to the US 1 h after training diminishes the learned approach in parallel behavioral experiments, it did not eliminate the memory trace in the β′2 MBONs. These findings suggest that, rather than an erasure of the reward-memory trace, US re-exposure introduces a long-lasting interference that prevents memory expression. In a study on aversive LTM in the fly, distracting stimuli, e.g., airflow or blue light, leads to a transient lack of memory availability without affecting memory traces measured in the dendrites of MBONs.[32] Thus, interference with memory expression might act downstream of the KC to MBON synapses. Given that US exposure before the training does not change approach behavior in our data, interference seems to act specifically on existing memories. This specificity could arise from the formation of antagonistic memories, as observed during extinction learning.[42,43] Here, the re-exposure to the CS+ alone does not change the initial memory trace but leads to the formation of parallel memory traces in distinct MBONs.[43,59] In this scenario, the lack of retrieval reflects the integration of newly formed inhibitory associations with pre-existing excitatory memory representations. Our findings revealed that most of the canonical MB circuits are not involved in the US-induced memory devaluation. This allows the speculation that the US-induced interference is established in circuits outside the MB, e.g., in the lateral horn. However, future experiments are required to understand where and how such interference is established to mediate US-driven memory devaluation.

Context-dependent memory re-evaluation

Memory re-evaluation processes such as extinction and reconsolidation critically depend on detecting a mismatch, or prediction error, during memory retrieval.[60–62] Although the role of context in extinction memory has been thoroughly explored,[4] little is known about how contextual cues influence other re-evaluation mechanisms, including reconsolidation. Context can be defined as a variable that depends on sensory inputs, internal states, and behavioral actions that modulate how memories are retrieved and expressed.[48] Our findings demonstrate that the devaluation of appetitive olfactory memories via US re-exposure is context dependent. Memory devaluation is robust when flies re-encounter the US in housing vials that resemble their housing or starvation environments. In contrast, re-exposure to the same US in the training apparatus (the T-maze) fails to alter memory expression. This suggests that the expectation of sugar availability, shaped by sensory input from a physical context, may determine the extent of memory updating, a concept that aligns with theories emphasizing the role of prediction error in memory modification.[60,62–64] Supporting this idea, recent studies in rats have shown that the magnitude of the prediction error during re-exposure governs how contextual information is integrated into memory re-evaluation.[65] In our work we showed that new learning, the pairing of an odor with the re-exposure to the US in housing vials, can prevent memory devaluation. This protection from devaluation suggests that new learning provides a boundary condition for memory devaluation, possibly by altering the perceived context. Such protection from memory update by new learning is in line with previous research on memory reconsolidation, which shows that these two processes, new learning and memory revaluation, are mutually exclusive.[10] Together, our experiments show that contextual information gate memory devaluation. Interestingly, the changes induced by artificially activating DANs appear to be context independent. This divergence suggests that not all memory re-evaluation processes are equally influenced by contextual information. Understanding the difference between the two processes will help us to understand how contextual information can gate or promote memory devaluation. Like previous work in invertebrates, these insights into the basic principles of gating memory devaluation might help us to develop strategies to target maladaptive memories.

RESOURCE AVAILABILITY

Lead contact

Requests for further information and resources should be directed to, and will be fulfilled by, the lead contact, Johannes Felsenberg (johannes.felsenberg@fmi.ch).

Materials availability

This study did not generate new, unique reagents.

Data and code availability

- This study did not generate any large-scale datasets; all data supporting the findings of this study are available from the lead contact upon reasonable request.
- This study did not generate new custom code. Any analysis scripts used in this study are available from the lead contact upon request.
- Any additional information required to reanalyze the data reported in this paper is available from the lead contact upon request.

ACKNOWLEDGMENTS

We thank the Janelia FlyLight Project and the Bloomington Drosophila Stock Center for the flies, the FMI core facilities for providing support, Lisa Scheunemann and Emmanuelle Perisse for their discussion and feedback on the project, and the Felsenberg lab members for their contributions and help. C.W. is funded by the Boehringer Ingelheim Fonds PhD Fellowship. This project has received funding from the Swiss National Science Foundation, the Novartis Research Foundation, and the European Research Council (ERC).

AUTHOR CONTRIBUTIONS

Conceptualization, C.W. and J.F.; methodology, C.W., J.A.S., D.G., B.Z., and J.F.; investigation, C.W., J.A.S., B.Z., and K.L.; formal analysis, C.W., J.A.S.,

CellPress
OPEN ACCESS

D.G., and B.Z.; software, D.G. and B.Z.; validation, C.W., J.A.S., and K.L.; funding acquisition, C.W. and J.F.; writing – original draft, C.W. and J.F.; and writing – review and editing, C.W., J.A.S., B.Z., D.G., K.L., and J.F.

DECLARATION OF INTERESTS

The authors declare no competing interests.

STAR★METHODS

Detailed methods are provided in the online version of this paper and include the following:

- KEY RESOURCES TABLE
- EXPERIMENTAL MODEL AND STUDY PARTICIPANT DETAILS
- METHOD DETAILS
  - Behavioral Experiments
  - Pharmacology
  - Immunohistochemistry
  - Calcium Imaging recording
- QUANTIFICATION AND STATISTICAL ANALYSIS
  - Behavioral assays analysis
  - Calcium Imaging analysis

SUPPLEMENTAL INFORMATION

Supplemental information can be found online at https://doi.org/10.1016/j.cub.2025.11.058.

Received: March 21, 2025
Revised: July 27, 2025
Accepted: November 21, 2025
Published: December 19, 2025

REFERENCES

1. Kindt, M. (2014). A behavioural neuroscience perspective on the aetiology and treatment of anxiety disorders. Behav. Res. Ther. *62*, 24–36. https://doi.org/10.1016/j.brat.2014.08.012.
2. Milton, A.L., and Everitt, B.J. (2012). The persistence of maladaptive memory: addiction, drug memories and anti-relapse treatments. Neurosci. Biobehav. Rev. *36*, 1119–1139. https://doi.org/10.1016/j.neubiorev.2012.01.002.
3. Lee, J.L.C., Nader, K., and Schiller, D. (2017). An update on memory reconsolidation updating. Trends Cogn. Sci. *21*, 531–545. https://doi.org/10.1016/j.tics.2017.04.006.
4. Bouton, M.E., Maren, S., and McNally, G.P. (2021). Behavioral and neurobiological mechanisms of pavlovian and instrumental extinction learning. Physiol. Rev. *101*, 611–681. https://doi.org/10.1152/physrev.00016.2020.
5. Pavlov, P.I. (1927). Conditioned Reflexes: An Investigation of the Physiological Activity of the Cerebral Cortex (Oxford University Press).
6. McGaugh, J.L. (2000). Memory–a century of consolidation. Science *287*, 248–251. https://doi.org/10.1126/science.287.5451.248.
7. Squire, L.R., Genzel, L., Wixted, J.T., and Morris, R.G. (2015). Memory consolidation. Cold Spring Harb. Perspect. Biol. *7*, a021766. https://doi.org/10.1101/cshperspect.a021766.
8. Pedreira, M.E., and Maldonado, H. (2003). Protein synthesis subserves reconsolidation or extinction depending on reminder duration. Neuron *38*, 863–869. https://doi.org/10.1016/S0896-6273(03)00352-0.
9. Nader, K., Schafe, G.E., and Le Doux, J.E. (2000). Fear memories require protein synthesis in the amygdala for reconsolidation after retrieval. Nature *406*, 722–726. https://doi.org/10.1038/35021052.
10. Merlo, E., Milton, A.L., Goozée, Z.Y., Theobald, D.E., and Everitt, B.J. (2014). Reconsolidation and extinction are dissociable and mutually exclusive processes: behavioral and molecular evidence. J. Neurosci. *34*, 2422–2431. https://doi.org/10.1523/jneurosci.4001-13.2014.
11. Debiec, J., Diaz-Mataix, L., Bush, D.E.A., Doyère, V., and LeDoux, J.E. (2013). The selectivity of aversive memory reconsolidation and extinction processes depends on the initial encoding of the Pavlovian association. Learn. Mem. *20*, 695–699. https://doi.org/10.1101/lm.031609.113.
12. Liu, J., Zhao, L., Xue, Y., Shi, J., Suo, L., Luo, Y., Chai, B., Yang, C., Fang, Q., Zhang, Y., et al. (2014). An unconditioned stimulus retrieval extinction procedure to prevent the return of fear memory. Biol. Psychiatry *76*, 895–901. https://doi.org/10.1016/j.biopsych.2014.03.027.
13. Teng, S.-W., Wang, X.-R., Du, B.-W., Chen, X.-L., Fu, G.-Z., Liu, Y.-F., Xu, S.-Q., Shuai, J.-C., and Chen, Z.-Y. (2023). Altered fear engram encoding underlying conditioned versus unconditioned stimulus–initiated memory updating. Sci. Adv. *9*, eadf0284. https://doi.org/10.1126/sciadv.adf0284.
14. Krashes, M.J., and Waddell, S. (2008). Rapid consolidation to a radish and protein synthesis-dependent long-term memory after single-session appetitive olfactory conditioning in Drosophila. J. Neurosci. *28*, 3103–3113. https://doi.org/10.1523/jneurosci.5333-07.2008.
15. Tempel, B.L., Bonini, N., Dawson, D.R., and Quinn, W.G. (1983). Reward learning in normal and mutant Drosophila. Proc. Natl. Acad. Sci. USA *80*, 1482–1486. https://doi.org/10.1073/pnas.80.5.1482.
16. Heisenberg, M. (2003). Mushroom body memoir: from maps to models. Nat. Rev. Neurosci. *4*, 266–275. https://doi.org/10.1038/nrn1074.
17. Cognigni, P., Felsenberg, J., and Waddell, S. (2018). Do the right thing: neural network mechanisms of memory formation, expression and update in Drosophila. Curr. Opin. Neurobiol. *49*, 51–58. https://doi.org/10.1016/j.conb.2017.12.002.
18. Aso, Y., Siwanowicz, I., Bräcker, L., Ito, K., Kitamoto, T., and Tanimoto, H. (2010). Specific dopaminergic neurons for the formation of labile aversive memory. Curr. Biol. *20*, 1445–1451. https://doi.org/10.1016/j.cub.2010.06.048.
19. Claridge-Chang, A., Roorda, R.D., Vrontou, E., Sjulson, L., Li, H., Hirsh, J., and Miesenböck, G. (2009). Writing memories with light-addressable reinforcement circuitry. Cell *139*, 405–415. https://doi.org/10.1016/j.cell.2009.08.034.
20. Yamagata, N., Ichinose, T., Aso, Y., Plaçais, P.Y., Friedrich, A.B., Sima, R.J., Preat, T., Rubin, G.M., and Tanimoto, H. (2015). Distinct dopamine neurons mediate reward signals for short- and long-term memories. Proc. Natl. Acad. Sci. USA *112*, 578–583. https://doi.org/10.1073/pnas.1421930112.
21. Huetteroth, W., Perisse, E., Lin, S., Klappenbach, M., Burke, C., and Waddell, S. (2015). Sweet taste and nutrient value subdivide rewarding dopaminergic neurons in Drosophila. Curr. Biol. *25*, 751–758. https://doi.org/10.1016/j.cub.2015.01.036.
22. Ichinose, T., Aso, Y., Yamagata, N., Abe, A., Rubin, G.M., and Tanimoto, H. (2015). Reward signal in a recurrent circuit drives appetitive long-term memory formation. eLife *4*, e10719. https://doi.org/10.7554/eLife.10719.
23. Liu, C., Plaçais, P.Y., Yamagata, N., Pfeiffer, B.D., Aso, Y., Friedrich, A.B., Siwanowicz, I., Rubin, G.M., Preat, T., and Tanimoto, H. (2012). A subset of dopamine neurons signals reward for odour memory in Drosophila. Nature *488*, 512–516. https://doi.org/10.1038/nature11304.
24. Burke, C.J., Huetteroth, W., Owald, D., Perisse, E., Krashes, M.J., Das, G., Gohl, D., Silies, M., Certel, S., and Waddell, S. (2012). Layered reward signalling through octopamine and dopamine in Drosophila. Nature *492*, 433–437. https://doi.org/10.1038/nature11614.
25. Trannoy, S., Redt-Clouet, C., Dura, J.M., and Preat, T. (2011). Parallel processing of appetitive short- and long-term memories in Drosophila. Curr. Biol. *21*, 1647–1653. https://doi.org/10.1016/j.cub.2011.08.032.
26. Bouzaiane, E., Trannoy, S., Scheunemann, L., Plaçais, P.Y., and Preat, T. (2015). Two independent mushroom body output circuits retrieve the six discrete components of Drosophila aversive memory. Cell Rep. *11*, 1280–1292. https://doi.org/10.1016/j.celrep.2015.04.044.
27. Cervantes-Sandoval, I., Martin-Peña, A., Berry, J.A., and Davis, R.L. (2013). System-like consolidation of olfactory memories in Drosophila.

J. Neurosci. *33*, 9846–9854. https://doi.org/10.1523/jneurosci.0451-13.2013.

28. Krashes, M.J., Keene, A.C., Leung, B., Armstrong, J.D., and Waddell, S. (2007). Sequential use of mushroom body neuron subsets during Drosophila odor memory processing. Neuron *53*, 103–115. https://doi.org/10.1016/j.neuron.2006.11.021.
29. Owald, D., Felsenberg, J., Talbot, C.B., Das, G., Perisse, E., Huetteroth, W., and Waddell, S. (2015). Activity of defined mushroom body output neurons underlies learned olfactory behavior in Drosophila. Neuron *86*, 417–427. https://doi.org/10.1016/j.neuron.2015.03.025.
30. Plaçais, P.Y., Trannoy, S., Friedrich, A.B., Tanimoto, H., and Preat, T. (2013). Two pairs of mushroom body efferent neurons are required for appetitive long-term memory retrieval in Drosophila. Cell Rep. *5*, 769–780. https://doi.org/10.1016/j.celrep.2013.09.032.
31. Berry, J.A., Cervantes-Sandoval, I., Nicholas, E.P., and Davis, R.L. (2012). Dopamine is required for learning and forgetting in Drosophila. Neuron *74*, 530–542. https://doi.org/10.1016/j.neuron.2012.04.007.
32. Sabandal, J.M., Berry, J.A., and Davis, R.L. (2021). Dopamine-based mechanism for transient forgetting. Nature *591*, 426–430. https://doi.org/10.1038/s41586-020-03154-y.
33. Siju, K.P., Štih, V., Aimon, S., Gjorgjieva, J., Portugues, R., and Grunwald Kadow, I.C. (2020). Valence and state-dependent population coding in dopaminergic neurons in the fly mushroom body. Curr. Biol. *30*, 2104–2115.e4. https://doi.org/10.1016/j.cub.2020.04.037.
34. Cohn, R., Morantte, I., and Ruta, V. (2015). Coordinated and compartmentalized neuromodulation shapes sensory processing in Drosophila. Cell *163*, 1742–1755. https://doi.org/10.1016/j.cell.2015.11.019.
35. Krashes, M.J., DasGupta, S., Vreede, A., White, B., Armstrong, J.D., and Waddell, S. (2009). A neural circuit mechanism integrating motivational state with memory expression in Drosophila. Cell *139*, 416–427. https://doi.org/10.1016/j.cell.2009.08.035.
36. Senapati, B., Tsao, C.H., Juan, Y.A., Chiu, T.H., Wu, C.L., Waddell, S., and Lin, S. (2019). A neural mechanism for deprivation state-specific expression of relevant memories in Drosophila. Nat. Neurosci. *22*, 2029–2039. https://doi.org/10.1038/s41593-019-0515-z.
37. Sayin, S., De Backer, J.F., Siju, K.P., Wosniack, M.E., Lewis, L.P., Frisch, L.M., Gansen, B., Schlegel, P., Edmondson-Stait, A., Sharifi, N., et al. (2019). A neural circuit arbitrates between persistence and withdrawal in hungry Drosophila. Neuron *104*, 544–558.e6. https://doi.org/10.1016/j.neuron.2019.07.028.
38. Aso, Y., and Rubin, G.M. (2016). Dopaminergic neurons write and update memories with cell-type-specific rules. eLife *5*, e16135. https://doi.org/10.7554/eLife.16135.
39. Burke, C.J., and Waddell, S. (2011). Remembering nutrient quality of sugar in Drosophila. Curr. Biol. *21*, 746–750. https://doi.org/10.1016/j.cub.2011.03.032.
40. Aso, Y., Ray, R.P., Long, X., Bushey, D., Cichewicz, K., Ngo, T.-T.B., Sharp, B., Christoforou, C., Hu, A., Lemire, A.L., et al. (2019). Nitric oxide acts as a cotransmitter in a subset of dopaminergic neurons to diversify memory dynamics. eLife *8*, e49257. https://doi.org/10.7554/eLife.49257.
41. Berry, J.A., Cervantes-Sandoval, I., Chakraborty, M., and Davis, R.L. (2015). Sleep facilitates memory by blocking dopamine neuron-mediated forgetting. Cell *161*, 1656–1667. https://doi.org/10.1016/j.cell.2015.05.027.
42. Felsenberg, J., Barnstedt, O., Cognigni, P., Lin, S., and Waddell, S. (2017). Re-evaluation of learned information in Drosophila. Nature *544*, 240–244. https://doi.org/10.1038/nature21716.
43. Felsenberg, J., Jacob, P.F., Walker, T., Barnstedt, O., Edmondson-Stait, A.J., Pleijzier, M.W., Otto, N., Schlegel, P., Sharifi, N., Perisse, E., et al. (2018). Integration of parallel opposing memories underlies memory extinction. Cell *175*, 709–722.e15. https://doi.org/10.1016/j.cell.2018.08.021.
44. Yamazaki, D., Maeyama, Y., and Tabata, T. (2023). Combinatory actions of co-transmitters in dopaminergic systems modulate Drosophila olfactory memories. J. Neurosci. *43*, 8294–8305. https://doi.org/10.1523/JNEUROSCI.2152-22.2023.
45. Musso, P.Y., Lampin-Saint-Amaux, A., Tchenio, P., and Preat, T. (2017). Ingestion of artificial sweeteners leads to caloric frustration memory in Drosophila. Nat. Commun. *8*, 1803. https://doi.org/10.1038/s41467-017-01989-0.
46. Kitamoto, T. (2001). Conditional modification of behavior in Drosophila by targeted expression of a temperature-sensitive shibire allele in defined neurons. J. Neurobiol. *47*, 81–92. https://doi.org/10.1002/neu.1018.
47. Dana, H., Sun, Y., Mohar, B., Hulse, B.K., Kerlin, A.M., Hasseman, J.P., Tsegaye, G., Tsang, A., Wong, A., Patel, R., et al. (2019). High-performance calcium sensors for imaging activity in neuronal populations and microcompartments. Nat. Methods *16*, 649–657. https://doi.org/10.1038/s41592-019-0435-6.
48. Heald, J.B., Lengyel, M., and Wolpert, D.M. (2023). Contextual inference in learning and memory. Trends Cogn. Sci. *27*, 43–64. https://doi.org/10.1016/j.tics.2022.10.004.
49. Stemerding, L.E., Stibbe, D., van Ast, V.A., and Kindt, M. (2022). Demarcating the boundary conditions of memory reconsolidation: An unsuccessful replication. Sci. Rep. *12*, 2285. https://doi.org/10.1038/s41598-022-06119-5.
50. Hige, T., Aso, Y., Modi, M.N., Rubin, G.M., and Turner, G.C. (2015). Heterosynaptic plasticity underlies aversive olfactory learning in Drosophila. Neuron *88*, 985–998. https://doi.org/10.1016/j.neuron.2015.11.003.
51. Dębiec, J., Díaz-Mataix, L., Bush, D.E.A., Doyère, V., and Ledoux, J.E. (2010). The amygdala encodes specific sensory features of an aversive reinforcer. Nat. Neurosci. *13*, 536–537. https://doi.org/10.1038/nn.2520.
52. Luo, Y.X., Xue, Y.X., Liu, J.F., Shi, H.S., Jian, M., Han, Y., Zhu, W.L., Bao, Y.P., Wu, P., Ding, Z.B., et al. (2015). A novel UCS memory retrieval-extinction procedure to inhibit relapse to drug seeking. Nat. Commun. *6*, 7675. https://doi.org/10.1038/ncomms8675.
53. Handler, A., Graham, T.G.W., Cohn, R., Morantte, I., Siliciano, A.F., Zeng, J., Li, Y., and Ruta, V. (2019). Distinct dopamine receptor pathways underlie the temporal sensitivity of associative learning. Cell *178*, 60–75.e19. https://doi.org/10.1016/j.cell.2019.05.040.
54. Yamagata, N., Hiroi, M., Kondo, S., Abe, A., and Tanimoto, H. (2016). Suppression of dopamine neurons mediates reward. PLoS Biol. *14*, e1002586. https://doi.org/10.1371/journal.pbio.1002586.
55. Pardo-Garcia, T.R., Gu, K., Woerner, R.K.R., and Dus, M. (2023). Food memory circuits regulate eating and energy balance. Curr. Biol. *33*, 215–227.e3. https://doi.org/10.1016/j.cub.2022.11.039.
56. Tsao, C.-H., Chen, C.-C., Lin, C.-H., Yang, H.-Y., and Lin, S. (2018). Drosophila mushroom bodies integrate hunger and satiety signals to control innate food-seeking behavior. eLife *7*, e35264. https://doi.org/10.7554/eLife.35264.
57. Musso, P.Y., Tchenio, P., and Preat, T. (2015). Delayed dopamine signaling of energy level builds appetitive long-term memory in Drosophila. Cell Rep. *10*, 1023–1031. https://doi.org/10.1016/j.celrep.2015.01.036.
58. Pribbenow, C., Chen, Y.-C., Heim, M.-M., Laber, D., Reubold, S., Reynolds, E., Balles, I., Fernández-D V Alquicira, T., Suárez-Grimalt, R., Scheunemann, L., et al. (2022). Postsynaptic plasticity of cholinergic synapses underlies the induction and expression of appetitive and familiarity memories in Drosophila. eLife *11*, e80445. https://doi.org/10.7554/eLife.80445.
59. Yang, Q., Zhou, J., Wang, L., Hu, W., Zhong, Y., and Li, Q. (2023). Spontaneous recovery of reward memory through active forgetting of extinction memory. Curr. Biol. *33*, 838–848.e3. , e833. https://doi.org/10.1016/j.cub.2023.01.022.
60. Fernández, R.S., Boccia, M.M., and Pedreira, M.E. (2016). The fate of memory: reconsolidation and the case of prediction error. Neurosci. Biobehav. Rev. *68*, 423–441. https://doi.org/10.1016/j.neubiorev.2016.06.004.

61. Cahill, E.N., Wood, M.A., Everitt, B.J., and Milton, A.L. (2019). The role of prediction error and memory destabilization in extinction of cued-fear within the reconsolidation window. Neuropsychopharmacology *44*, 1762–1768. https://doi.org/10.1038/s41386-018-0299-y.

62. Milton, A.L., Das, R.K., and Merlo, E. (2023). The challenge of memory destabilisation: From prediction error to prior expectations and biomarkers. Brain Res. Bull. *194*, 100–104. https://doi.org/10.1016/j.brainresbull.2023.01.010.

63. Gershman, S.J., Monfils, M.-H., Norman, K.A., and Niv, Y. (2017). The computational nature of memory modification. eLife *6*, e23763. https://doi.org/10.7554/eLife.23763.

64. Cochran, A.L., and Cisler, J.M. (2019). A flexible and generalizable model of online latent-state learning. PLoS Comput. Biol. *15*, e1007331. https://doi.org/10.1371/journal.pcbi.1007331.

65. Kennedy, N.G.W., Lee, J.C., Killcross, S., Westbrook, R.F., and Holmes, N.M. (2024). Prediction error determines how memories are organized in the brain. eLife *13*, RP95849. https://doi.org/10.7554/elife.95849.

66. Schindelin, J., Arganda-Carreras, I., Frise, E., Kaynig, V., Longair, M., Pietzsch, T., Preibisch, S., Rueden, C., Saalfeld, S., Schmid, B., et al. (2012). Fiji: an open-source platform for biological-image analysis. Nat. Methods *9*, 676–682. https://doi.org/10.1038/nmeth.2019.

67. Leinweber, M., Zmarz, P., Buchmann, P., Argast, P., Hübener, M., Bonhoeffer, T., and Keller, G.B. (2014). Two-photon calcium imaging in mice navigating a virtual reality environment. J. Vis. Exp, e50885. https://doi.org/10.3791/50885.

68. Štih, V., Petrucco, L., Kist, A.M., and Portugues, R. (2019). Stytra: An open-source, integrated system for stimulation, tracking and closed-loop behavioral experiments. PLoS Comput. Biol. *15*, e1006699. https://doi.org/10.1371/journal.pcbi.1006699.

69. Giovannucci, A., Friedrich, J., Gunn, P., Kalfon, J., Brown, B.L., Koay, S.A., Taxidis, J., Najafi, F., Gauthier, J.L., Zhou, P., et al. (2019). CaImAn an open source tool for scalable calcium imaging data analysis. eLife *8*, e38173. https://doi.org/10.7554/eLife.38173.

STAR★METHODS

KEY RESOURCES TABLE

REAGENT or RESOURCE	SOURCE	IDENTIFIER
Antibodies		
Anti GFP (chicken)	Abcam	ab13970; RRID: AB_300798
Anti Bruchpilot (mouse)	DSHB	nc82; RRID: AB_2314866
Goat anti-Chicken IgY (H+L) Alexa Fluor 488	Invitrogen	A-11039; RRID: AB_2534096
Goat anti-Mouse IgG (H+L) Alexa Fluor 633	Invitrogen	A-21052; RRID: AB_2535719
Chemicals, peptides, and recombinant proteins		
4-methylcyclohexanol (98%)	Sigma-Aldrich	Cat#153095
3-octanol (99%)	Sigma-Aldrich	Cat#218405
ethyl butyrate (EB)	Sigma-Aldrich	Cat#E15701
isopentyl acetate (IPA)	Sigma-Aldrich	Cat#112674
All trans-Retinal	Sigma-Aldrich	Cat#R2500
L-NNA (Nω-Nitro-L-Arginin)	Sigma-Aldrich	Cat#N5501
Experimental models: Organisms/strains		
D. melanogaster: Canton S	Waddell lab, University of Oxford	N/A
D. melanogaster: R58E02-Gal4	Bloomington Drosophila Stock Center	RRID: BDSC_41347
D. melanogaster: R58E02-lexA	Bloomington Drosophila Stock Center	RRID: BDSC_52740
D. melanogaster: R48B04-Gal4	Bloomington Drosophila Stock Center	RRID: BDSC_50347
D. melanogaster: R15A04-Gal4	Bloomington Drosophila Stock Center	RRID: BDSC_48671
D. melanogaster: VT1211-Gal4	Vienna *Drosophila* Resource Center	v202324
D. melanogaster: R13F02-Gal4	Bloomington Drosophila Stock Center	RRID: BDSC_48571
D. melanogaster: Tcd2-Gal4	Bloomington Drosophila Stock Center	RRID: BDSC_9313
D. melanogaster: Trhn-Gal4	Bloomington Drosophila Stock Center	RRID: BDSC_38388
D. melanogaster: 5015(DPM)-Gal4	Bloomington Drosophila Stock Center	RRID: BDSC_2721
D. melanogaster: LH989-Gal4/ SS04956-Gal4	Bloomington Drosophila Stock Center	RRID: BDSC_86697
D. melanogaster: MB194B-Gal4	Bloomington Drosophila Stock Center	RRID: BDSC_68269
D. melanogaster: MB043C-Gal4	Bloomington Drosophila Stock Center	RRID: BDSC_68363
D. melanogaster: Gr64f-Gal4	Bloomington Drosophila Stock Center	RRID: BDSC_57669
D. melanogaster: repo-Gal4	Bloomington Drosophila Stock Center	RRID: BDSC_7415
D. melanogaster: empty-Gal4	Bloomington Drosophila Stock Center	RRID: BDSC_68384
D. melanogaster: empty split-Gal4	Bloomington Drosophila Stock Center	RRID: BDSC_79603
D. melanogaster: UAS-*shi*^{ts}	Waddell lab, University of Oxford	N/A
D. melanogaster: UAS-CsChrimson	Waddell lab, University of Oxford	N/A
D. melanogaster: UAS-dTRPA1	Bloomington Drosophila Stock Center	RRID: BDSC_26263
D. melanogaster: UAS-dTRPA1, lexAOP-CsChrimson; R58E02-Gal4	This study	N/A
D. melanogaster: UAS-jGCaMP7s	Bloomington Drosophila Stock Center	RRID:BDSC_79032
D. melanogaster: UAS-RSET-jGCaMP8f	Bloomington Drosophila Stock Center	RRID:BDSC_605085
Software and algorithms		
GraphPad Prism version 8.3.0 to 10.2.2	GraphPad Software, La Jolla, CA	N/A
Fiji	NIH; Schindelin et al.[66]	N/A
Adobe Illustrator CC	Adobe Systems, San Jose, CA	N/A

EXPERIMENTAL MODEL AND STUDY PARTICIPANT DETAILS

Drosophila melanogaster strains were reared and kept on standard cornmeal-agar food either at 60% humidity and 22°C or 25°C in a 12:12 h light-dark cycle. As wildtype strain Canton S flies were used. All other lines are listed in the key resources table and in Figure S4. Flies expressing *CsChrimson* were kept and handled in the dark or under dim light conditions and flipped to food containing 125 mM all-trans retinal 3 days prior to the experiment. Males from GAL4 or LexA driver lines were crossed to virgins from the UAS or lexAOP lines.

METHOD DETAILS

Behavioral Experiments

For behavioral experiments mixed-sex populations of 2–8-day old flies were used. Approximately 80-100 flies were starved for 16–20 h in a 25 ml vial (housing vial) containing 3 ml of 1% agar as water source and a 15 x 30 mm filter paper. As odors 4-methylcyclohexanol (MCH), 3-octanol (OCT), ethyl butyrate (EB) and isopentyl acetate (IPA) diluted approximately 10^{-3} in mineral oil were used. To investigate the effects of other odor concentrations OCT and MCH were used at a concentration of 2×10^{-3}. Standard experiments were performed at 23°C and 60% humidity. For thermogenetic activation of neurons by *dTRPA1* or inhibition by shi^{ts} temperature was raised to 32°C. In experiments using shi^{ts} flies were exposed to the restrictive temperature 30 min before the respective experimental phase.

Appetitive training was performed as described before.[14,29] Flies were exposed to the unpaired odor for 2 min without sucrose-reward, followed by 30 s of clean air. Subsequently flies encountered the paired odor for 2 min together with dry sucrose.

To activate *dTRPA1* expressing neurons flies were flipped to preheated (32°C) vials. For post-training activation flies remained in 32°C for 30 min before being transferred to room temperature. For optogenetic activation using *CsChrimson*, flies were exposed to red light (λ=634 nm, 500 Hz, 20 mW/cm^2).

Sugar re-exposure was done for 5 min either in 25 ml vials containing 3 ml of 1% agar or in T-maze tubes. To present the sugar vials/tubes were lined with a filter paper covered in dry sucrose. To control the effects of the handling, in parallel to flies that were re-exposed to sugar, a control group of flies were exposed to vials/tubes that contained just the filter paper. In all experiments the groups were run in parallel and the sequence of the two groups in each experimental run was alternated.

For the test flies were given the option between two odors in the T-maze. Testing was performed in the dark for 2 min. Afterwards flies were collected from either side of the T-maze and counted accordingly. Performance index was calculated as the difference between number of flies approaching the paired odor and the unpaired odor, divided by the total number of flies. One *n* consists of two sets of flies trained reciprocally in terms of odor identity for CS- and CS+.

Pharmacology

NOS activity was inhibited by feeding flies L-NNA. L-NNA was either given in food containing 100 mM L-NNA or on filter papers soaked with saturated L-NNA solution in tap water.

Immunohistochemistry

Fly brains were fixed for 30 min in 4% formaldehyde after dissection. Blocking was done overnight at 4°C with 5% goat serum in PBS containing 0.3% Triton X (PBS-T). Afterwards brains were incubated with the primary antibodies (chicken anti GFP, Abcam (ab13970) (diluted 1:2000); mouse anti Bruchpilot, DSHB (nc82) (diluted 1:50)) for at least 5 h at RT, sufficiently rinsed in PBS-T and afterwards incubated with the secondary antibodies (diluted 1:300) (Goat anti-Chicken IgY (H+L) Alexa Fluor 488; Goat anti-Mouse IgG (H+L) Alexa Fluor 633) overnight at 4°C. For mounting Vectashield mounting medium was used. Imaging was done with a Zeiss Axio Imager M2 equipped with a spinning disk confocal scanning unit (Yokogawa CSU W1 with Dual T2, Pinhole size: 50um) using a 20x air objective. Images were analyzed with Fiji.[66] Expression patterns are displayed in Figure S5.

Calcium Imaging recording

For imaging experiments, 3–8-day old female and male flies were briefly anesthetized on ice for 10 s and mounted in a custom-made 3D printed platform. The proboscis was restrained to minimize movements during recordings. The head capsule was immersed and opened in buffer solution at room temperature (5 mM TES, 103 mM NaCl, 3 mM KCl, 1.5 mM $CaCl_2$, 4 mM $MgCl_2$, 26 mM $NaHCO_3$, 1 mM NaH_2PO_4, 8 mM Trehalose, 10 mM glucose, pH 7). Membranes and trachea above the recording site were surgically removed. Head-fixed flies were placed under a modified Thorlabs Bergamo II two-photon microscope. A constant air stream at 0.9 L/min was directly applied to the antenna of the fly using an olfactometer (220A Aurora Scientific). For all flies, GCaMP7s fluorescence signal was measured in an arbitrarily chosen brain hemisphere. Fluorescence was excited using a Ti-Sapphire laser centered at 920 nm. Laser power under the objective was set between 10 to 25 mW, depending on the sample. Images of 750 x 400 pixels (approximately 300 μm x 300 μm) were acquired at a frequency of 60 Hz using custom software.[67] Stimuli delivery and synchronized imaging acquisition were controlled using Stytra.[68] Flies were exposed to a 5-second-long air puff twice at the beginning of each recording session with an inter-trial interval (ITI) of 30 s. After that, the unpaired and the paired odor were presented to the animal. The two odors were alternated and presented two times. Each odor presentation lasted 5 s and was followed by an ITI of 30 s during which clean air was provided. The three groups (mock, trained and sugar exposed flies) were exposed to the same protocol.

To record calcium responses in projections of PAM-DANs to the horizontal lobe of the MB, flies expressing GCaMP8f under the control of the driver R58E02 were utilized. Flies were either sucrose trained in the T-maze or exposed to odors only (mock trained) 2.5 h–3.5 h later flies were mounted in a custom-made 3D printed platform. The proboscis was fixed in a non-extended position using low melting paraffin while ensuring that the labellum was able to open. The front legs of the fly were fixed to the stage to ensure sucrose stimulation to the proboscis only. A 2 M water-based sucrose solution was colored with the food coloring dye Indigo carmine (Sigma Aldrich – 131164) and loaded into a pipette tip fixed to a custom-made automated fly feeder. Head capsule preparation was performed as mentioned above. Fluorescence in the horizontal lobe was recorded for 30 s before offering the sucrose to the fly by immersing the proboscis in the solution for 60 s. Only flies with a blue abdomen, proofing food ingestion were included for analysis.

QUANTIFICATION AND STATISTICAL ANALYSIS

Behavioral assays analysis

Data was analyzed with GraphPad Prism version 8.3.0 to 10.2.2. Groups were analyzed for Gaussian distribution using a Shapiro-Wilk normality test. For normally distributed data, a one-sample t-test was applied to check for significant positive or negative performance indexes (Significance level alpha = 0.05). Data sets containing two groups with normal distribution were analyzed for significant differences using an unpaired t-test. When data points were not normally distributed, a Mann-Whitney test was performed. One-way ANOVA tests were applied on data sets containing more than two groups. For correction of multiple comparisons in normally distributed data, a Tukey-Test was performed. Data sets with not normally distributed groups were analyzed using a Kruskal-Wallis test. For all tests, significance was defined as $P < 0.05$. Numbers of *n* for each experiment are mentioned in the corresponding figure legend.

Calcium Imaging analysis

Two-photon fluorescence images were manually segmented using the Python libraries EasyROI and OpenCV. When selecting the region of interest, background subtraction was performed. Movement correction was performed, after XY binning, using python library CaImAn.[69] The fluorescence over the defined region of interest was averaged at each frame to yield one fluorescence trace, F(t). Traces were then binned in time, reaching a final frequency of 4 Hz. All subsequent analyses were performed using custom-written Python notebooks. Baseline fluorescence (F0) corresponds to the average fluorescence signal during a 5-second window before each odor delivery. The baseline was then used to compute the relative change in fluorescence during the odor exposure ($\Delta F(t)/F_0 = (F(t) - F_0)/F_0$). We analyzed the data coherently with what has been done in the literature,[29] slightly adapting the formula for our system. For each fly *i* and each odor $CS^{+,-}$, the peak response during the 5 s of the odor delivery was normalized by subtracting the peak response to the air puff of the same individual and by normalizing to the peak response of the averaged peak response of the corresponding odor (paired or unpaired) of the mock group, following the formula:

$$\frac{[\mathrm{Peak}(\mathrm{CS}^{+,-})_i - \mathrm{Peak}(\mathrm{Puff})_i] - \langle \mathrm{Peak}(\mathrm{CS}^{+,-})\rangle_{\mathrm{mock}}}{\langle \mathrm{Peak}(\mathrm{CS}^{+,-})\rangle_{\mathrm{mock}}},$$

where

$$\langle \mathrm{Peak}(\mathrm{CS}^{+,-})\rangle_{\mathrm{mock}} = \frac{1}{N_{\mathrm{mock}}}\sum_{j=1}^{N_{\mathrm{mock}}}\left[\mathrm{Peak}(\mathrm{CS}^{+,-})_j - \mathrm{Peak}(\mathrm{Puff})_j\right].$$

Similarly, we normalized the traces by dividing each value of each trace of odor response to the average odor response of the same odor of the mock group, following the formula

$$\mathrm{CSnorm}_i(t) = \frac{\mathrm{CS}_i^{+,-}(t)}{\frac{1}{N_{\mathrm{mock}}}\sum_{j=1}^{N_{\mathrm{mock}}}\int_0^5 \mathrm{CS}_j^{+,-}(t)\,dt}.$$

Current Biology, Volume *36*

Supplemental Information

Re-exposure to reward

re-evaluates related memories

Carolin Warnecke, Johanna A. Schweizer, Benedetta Zattera, Dennis Goldschmidt, Kerstin Leptien, and Johannes Felsenberg

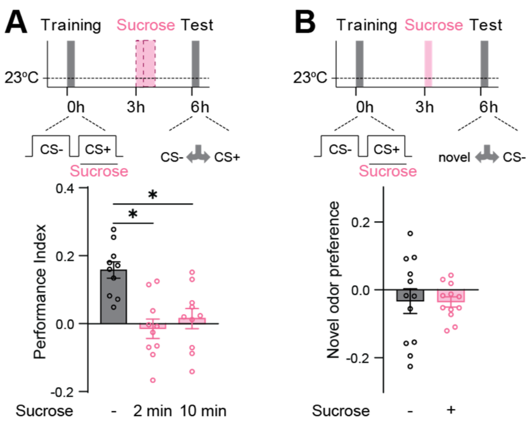

<u>Figure S1:</u> Reward re-exposure specifically diminishes expression of olfactory reward memory (related to Figure 1). A) Exposing flies to sucrose for 2 min or 10 min at 3 h after sucrose learning leads to diminished 6-h memory performance (*n* ≥ 9). B) Flies show no approach behavior to the CS- when given the choice between the CS- and a novel odor in a 6-h memory test, independently of whether they have been re-exposed to sugar 3 h after training or not (*n* ≥ 10). Asterisks denote significant differences ($p < 0.05$, ANOVA)

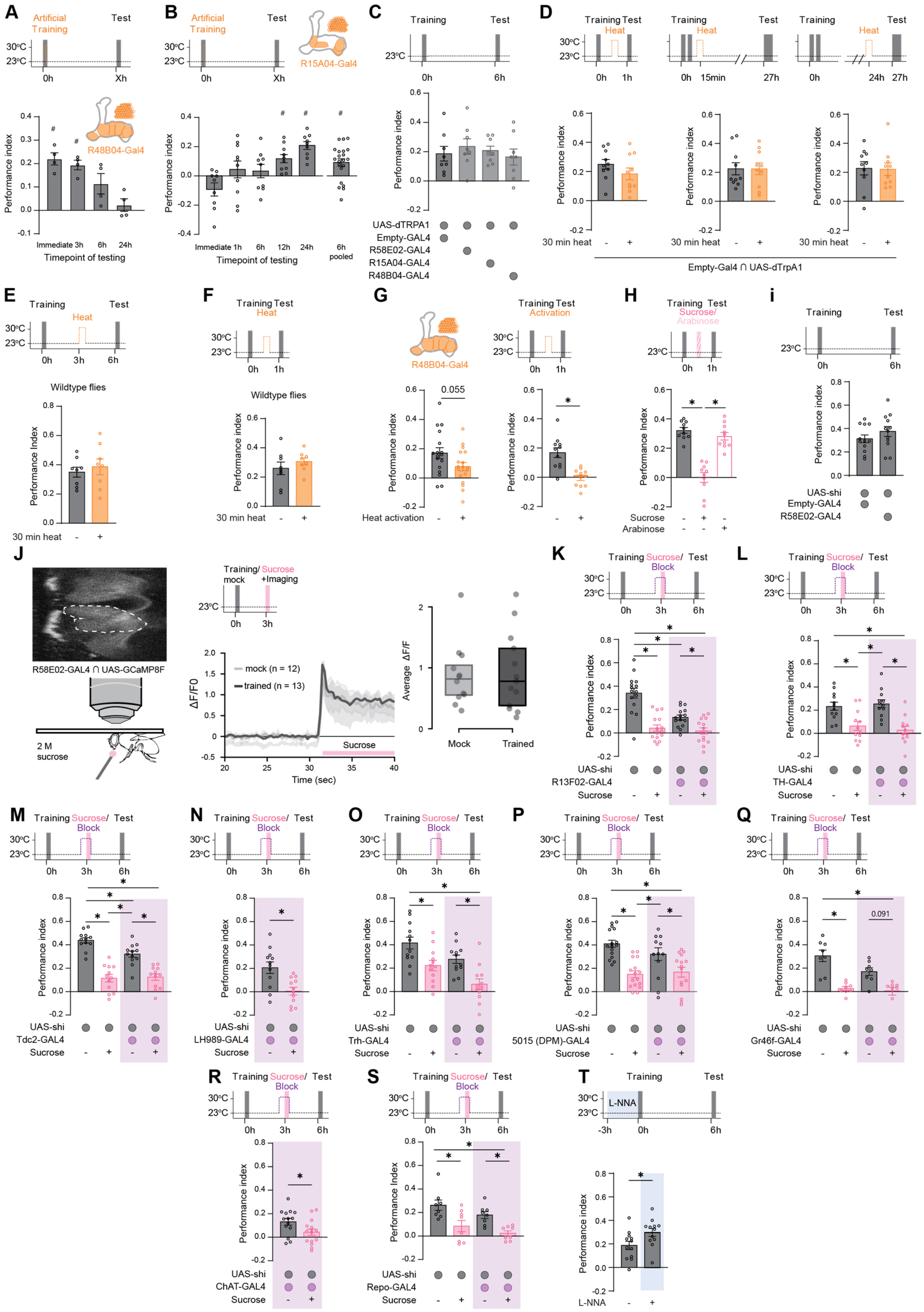

A
Artificial Training
Test
30ºC
23ºC
0h
Xh
R48B04-Gal4
Performance index
Immediate 3h 6h 24h
Timepoint of testing
B
R15A04-Gal4
Immediate 1h 6h 12h 24h 6h pooled
C
Training
Test
UAS-dTRPA1
Empty-GAL4
R58E02-GAL4
R15A04-GAL4
R48B04-GAL4
D
Heat
0h 1h
15min
27h
24h
30 min heat
Empty-Gal4 ∩ UAS-dTrpA1
E
Wildtype flies
Performance Index
F
G
Activation
Heat activation
0.055
H
Sucrose/ Arabinose
Sucrose
Arabinose
I
UAS-shi
Empty-GAL4
R58E02-GAL4
J
R58E02-GAL4 ∩ UAS-GCaMP8F
2 M sucrose
Training/ mock
Sucrose +Imaging
mock (n = 12)
trained (n = 13)
ΔF/F0
Time (sec)
Average ΔF/F
Mock
Trained
K
Training Sucrose/ Block Test
R13F02-GAL4
L
TH-GAL4
M
Tdc2-GAL4
N
LH989-GAL4
O
Trh-GAL4
P
5015 (DPM)-GAL4
Q
Gr46f-GAL4
0.091
R
ChAT-GAL4
S
Repo-GAL4
T
L-NNA
-3h

Figure S2: Activity of reward DANs bidirectionally influences sugar memory retrieval and sucrose-induced memory re-evaluation does not depend on PAM dopaminergic or other tested neural populations (related to Figure 2). A) Training with R48B04-GAL4 labeled neurons implements an appetitive STM (*n* =4). B) Artificial training with R15A04-GAL4 labeled neurons exclusively leads to appetitive LTM expression. A 6-h memory was not expressed in this data set. However, pooling 6-h memory performance with datapoints from other data sets shows robust memory expression (last bar "6 h pooled") (*n* ≥ 9). C) Flies expressing *UAS-TRPA1* in R58E02- GAL4, R15A04-GAL4 and R48B04-GAL4 labeled neurons show comparable performance in sucrose learning as controls crossed to empty-GAL4 (*n* ≥ 8). D) Flies expressing an UAS-TRPA1 together with an empty-GAL4 show no difference in memory performance when exposed to heat in the STM, LTM with early activation or LTM with late activation scheme (*n* ≥ 8). E) Exposing wild-type flies (wt, Canton-S) to 30 min heat 3 h after sucrose learning does not affect retrieval at the 6-h memory test (*n* ≥ 8). F) Exposing wt flies to heat for 30 min at 15 min after sucrose training does not influence 1-h memory retrieval (*n* ≥ 8). G) Activation of STM DANs 15 min after learning leads to diminished 1-h memory retrieval. The two graphs show the separate data of two repetitions of the same experiment. Combined data shown in Fig. 2D. H) Re-exposure to sucrose for 5 min at 30 min after sucrose training leads to diminished 1-h memory performance. Following the same timeline post-training exposure to arabinose has no effect on memory performance (*n* ≥ 8). I) Flies expressing shi^{ts} driven by R58E02-GAL4show similar memory performance after sucrose training compared to genetic controls (*n* ≥ 10). J) GCaMP8f expression in projection of PAM DANs under control of R58E02 driver. ROI depicted by dotted line. 3 h post training 2 M sucrose was offered to the proboscis of trained and mock trained flies placed under 2-photon microscope. Calcium signal was measured in the tip of the horizontal MB lobe. The averaged calcium signal in PAM dendrites in response to sucrose stimulation does not differ between trained and mock trained flies. K-S) Blocking any of the tested neuronal population during sucrose re-exposure does not impede sucrose-mediated memory devaluation. Candidates tested: KCs (R13F02-GAL4) (*n* ≥ 15) (K), PPL1-, PPL2-, PAL-, PPM1-3-DANs (TH-GAL4) (*n* ≥ 10) (L), octopaminergic neurons (Tdc2-GAL4) (*n* ≥ 12) (M), PD2a1/b1 LHONs (LH989-GAL4) (*n* ≥ 11) (N), serotonergic neurons (Trh-GAL4) (*n* ≥ 11) (O), pair of DPM-neurons (5015-GAL4) (*n* ≥ 12) (P), sweet gustatory receptor neurons (Gr64f-GAL4) (*n* ≥ 7) (Q), cholinergic neurons (ChAT-GAL4) (*n* ≥ 14) (R) and glia cells (repo-GAL4) (*n* ≥ 10) (S). T) Feeding L-NNA for 3 h before training to inhibit NOS activity leads to increased memory retrieval at a 6-h test (*n* ≥ 8). In all figures, data represent the mean ± s.e.m. Individual n are indicated by circles. Asterisks denote significant differences ($p < 0.05$, *t*-test or ANOVA). Asterisks denote significant differences to 0 ($p < 0.05$, one sample *t*-test) in A&B and unpaired *t*-test ($p < 0.05$) in G.

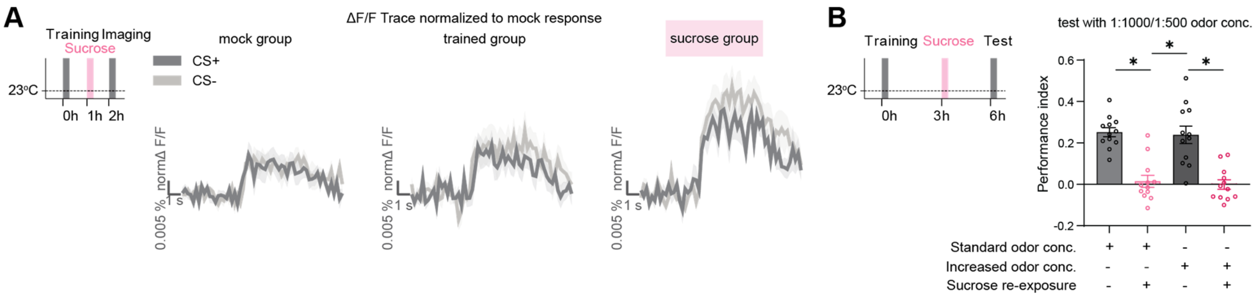

<u>Figure S3:</u> Memory trace is intact after US-re-exposure (related to Figure 3). A) Flies expressing GCamp7s in VT1211-Gal4 labeled neurons are sugar trained in T-mazes and re-exposed to sucrose in housing vials 1 h later, 2 h after learning calcium signals were measured in MBON-β'2mp and MBON-γ5β'2a neurons. Here, ΔF/F0 traces normalized to the average odor response of the corresponding odor of the mock are shown for the respective groups (see Methods). B) 6-h memory was tested after 5 min re-exposure to sucrose reward across different odor concentrations in the test ($n \geq 11$). Flies were trained with an odor concentration of $\sim 10^{-3}$ and tested with either the same concentration or a higher odor concentration ($2 \times \sim 10^{-3}$). Asterisks denote significant differences ($p < 0.05$, ANOVA).

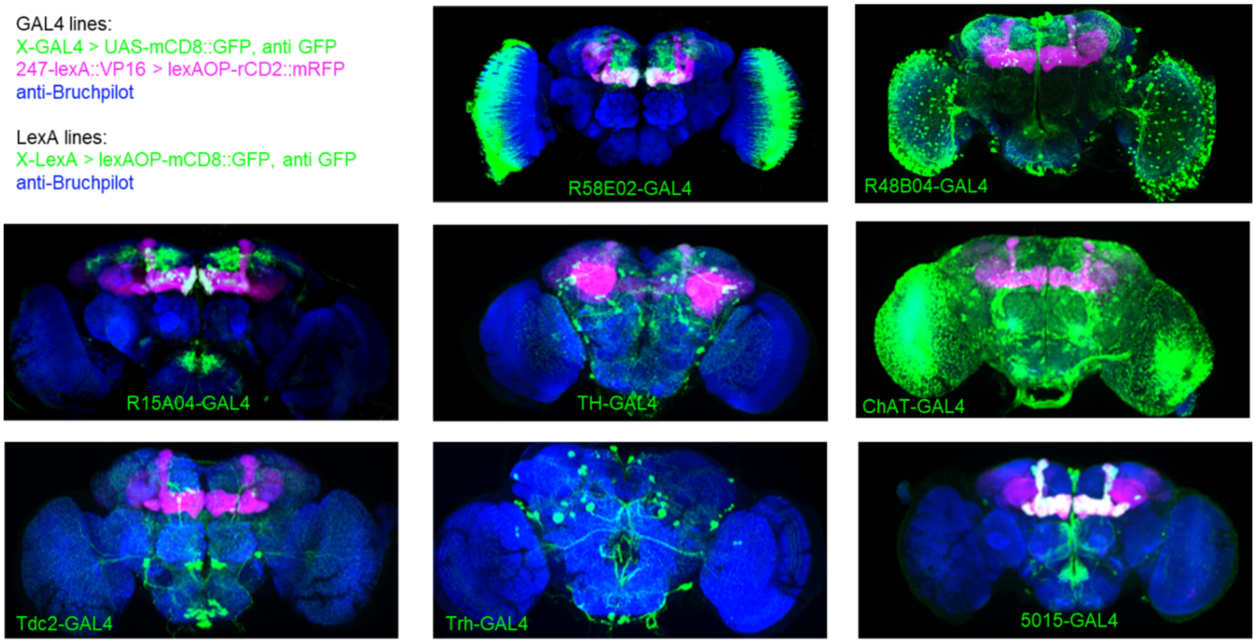

<u>Figure S4:</u> GAL4 expression patterns (related to Figure 2 and S2).
